# Translation factor eIF4G2 directs CD8^+^ T cell lineage commitment by selectively enabling the IL-7 receptor response

**DOI:** 10.1016/j.isci.2026.115313

**Published:** 2026-03-11

**Authors:** Jialong Cui, Xinhui Zhang, Yang Yang, Lidong Shan, Yang Li, Long Jiang, Wei Xie, Tengchuan Jin, Xueting Lang

**Affiliations:** 1 School of Basic Medical Sciences, Center for Big Data and Population Health of IHM, Anhui Medical University, Hefei, Anhui 230032, China; 2 Institute of Health and Medicine, Hefei Comprehensive National Science Center, Hefei, Anhui 230601, China

**Keywords:** Immunology, Cell biology

## Abstract

CD8^+^ T cell lineage commitment in the thymus requires interleukin-7 receptor (IL-7R) signaling, but the mechanisms enabling its cytokine responsiveness are unclear. Here, we identify the translation factor eIF4G2 as an essential, selective regulator of this process. eIF4G2 expression is upregulated in double-positive thymocytes and its T cell specific deletion causes a severe post-selection blockade, specifically abolishing CD8^+^ single positive thymocyte lineage commitment while sparing CD4^+^ lineage choice and TCR signaling. Mechanistically, eIF4G2 deficiency ablates IL-7 responsiveness by failing to sustain the receptor γc subunit via an untranslated region dependent manner, with a concomitant impairment of IL-7Rα mRNA level. Our findings establish eIF4G2 as a pivotal translational checkpoint that licenses IL-7R signaling to enforce faithful CD8^+^ T cell fate determination.

## Highlights

• Translational control governs CD8^+^ T cell lineage commitment
• eIF4G2 dictates IL-7 responsiveness
• eIF4G2 sustains surface expression of the IL-7 receptor
• eIF4G2 controls γc synthesis in a UTR-dependent manner

## Graphical abstract

**Figure:**
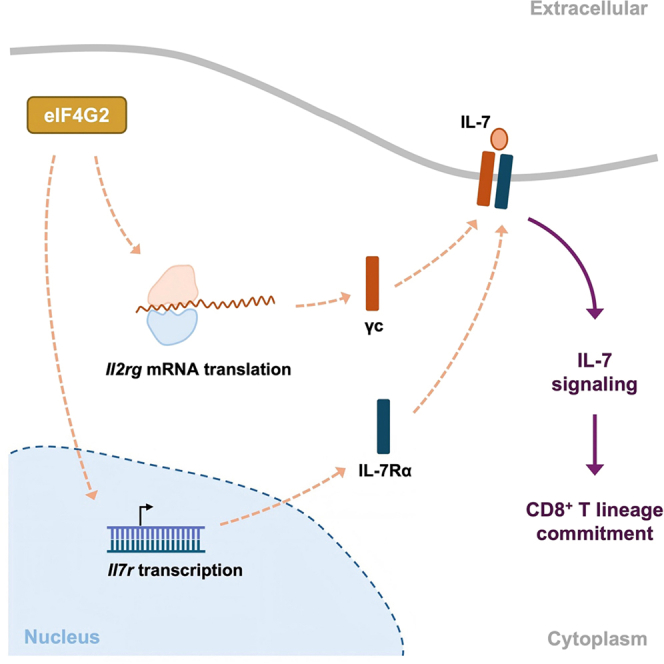

## Introduction

The generation of a diverse and self-tolerant T cell repertoire is required for the homeostasis maintenance and protection from disease in vertebrates.^1^ To ensure it, tightly regulated T cell development process is indispensable. There are several key stages during T cell development, including T cell progenitor generation in bone marrow, β selection in progenitor T cells, positive selection in CD4, and CD8 double-positive T cells (DP), as well as negative selection in immature single-positive T cells (SP).^2^ Among these stages, the signals received after positive selection in DP cells play a pivotal role in determining the differentiation direction and fate of T cells. Specifically, high strength and continuous TCR signaling is crucial for CD4^+^ T cell lineage commitment, whereas an efficient response to IL-7 cytokine is necessary for CD8^+^ T cell development.^3^ While the regulation of TCR signaling for CD4^+^ T cell fate determination is relatively well-characterized, the mechanisms ensuring a timely and effective IL-7 response to instruct CD8^+^ T cell fate are less clear.

The IL-7 receptor (IL-7R) complex, as the key transducer of IL-7 response, is a heterodimer of the unique IL-7Rα chain (IL-7Rα, encoded by *Il7r* in mice and *IL7R* in humans, also known as CD127) and the common γc chain (γc, encoded by *Il2rg* in mice and *IL2RG* in humans, also known as CD132).^4^^,^^5^ Efficient signal transduction through this receptor complex is indispensable for proper T cell development, especially CD8^+^ lineage commitment.^6^ However, the regulation of this receptor complex, specifically, the mechanisms that license it to mount a productive IL-7 response at the critical CD8^+^ lineage commitment stage, remains poorly understood. Current knowledge is largely confined to transcriptional regulators of the IL-7Rα subunit, which operate either in mature thymocytes to promote survival or in peripheral T cells to maintain homeostasis.^7^^,^^8^ It remains unknown whether and how distinct mechanisms endow the receptor complex with the signaling competence required to execute CD8^+^ lineage choice.

Translational control is a fundamental yet understudied layer of gene regulation in cell fate decisions. Eukaryotic translation initiation factors (eIFs), which orchestrate the rate-limiting step of translation initiation, are emerging as selective regulators of gene expression programs in diverse biological contexts, including oncogenesis and differentiation.^9^^,^^10^^,^^11^^,^^12^ However, whether eIFs contribute to T cell development, particularly to the specific T cell fate commitment stage, is largely unexplored.

Here, we identify the translation initiation factor eIF4G2 as an essential and specific regulator of CD8^+^ T cell lineage commitment. eIF4G2 is known for its roles in both canonical and alternative translation initiation and has been implicated in specialized cellular processes, including neuronal plasticity and stress responses,^13^^,^^14^^,^^15^^,^^16^^,^^17^ but its function in lymphocyte development was unknown. In this study, we report that T cell specific deletion of *Eif4g2* disrupts CD8^+^ lineage commitment by ablating IL-7 responsiveness. Mechanistically, eIF4G2 is required to maintain the IL-7 receptor complex by sustaining γc via UTRs-dependent translation, with IL-7Rα levels concomitantly reduced upon its loss. Our findings thus establish eIF4G2 as the pivotal translational checkpoint that licenses the IL-7 receptor complex, thereby establishing eIF4G2 as the factor that directs CD8^+^ T cell lineage commitment by selectively enabling the IL-7 receptor response.

## Results

### Result 1. *Eif4g2* expression is upregulated at late stage during T cell development

**Figure 1:**
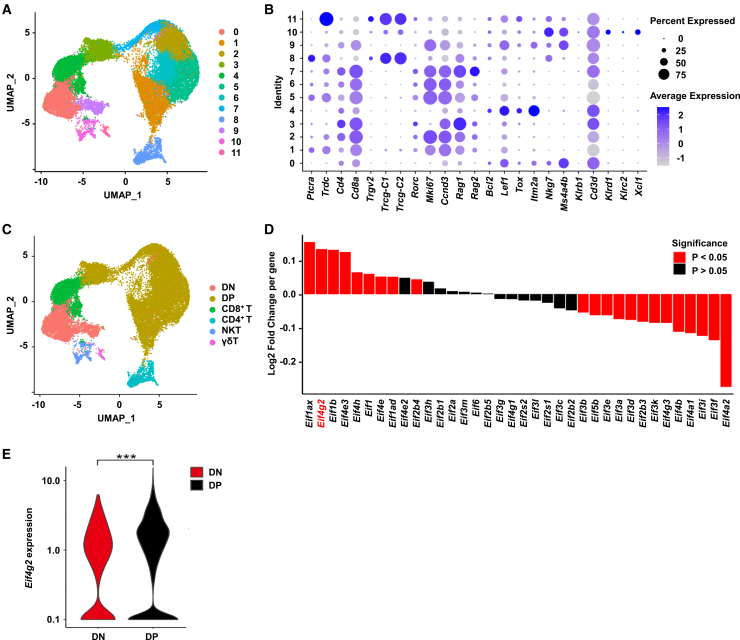
*Eif4g2* expression is upregulated at late stage during T cell development (A) UMAP dimensionality reduction of the first 25 PCs, classifying 12 cell clusters. (B) Dot plot showing marker genes used to identify clusters based on differential gene expression. (C) UMAP plot illustrating the subtypes of the T clusters, color-coded by cell type. (D) Expression ratio of translation initiation factor family members in DP and DN cells. Red bars indicate genes with *p* < 0.05, black bars indicate genes with *p* > 0.05. (E) Expression levels of *Eif4g2* in DN and DP cells. ∗∗∗*p* < 0.001. All data are plotted as mean ± SEM. See also Figure S1.

To investigate the potential roles of eIFs genes in T cell development, particularly at the late stages, we first analyzed their expression dynamics between DN and DP thymocytes using published mouse single-cell RNA sequencing data.^18^ The expression of several eIFs genes was significantly altered during this transition, with a subset being markedly upregulated at the DP stage (Figures 1A–1D and S1). Among the upregulated ones, eIF4G2 exhibited one of the most pronounced increases (Figures 1D and 1E). eIF4G2 is a unique translational factor known to function in both canonical cap-dependent and non-canonical cap-independent translation processes and has been reported to act as a selective regulator of gene expression under various physiological and pathophysiological context.^15^ Its dynamic upregulation during the DP stage prompted us to hypothesize that eIF4G2 might play a specialized role in late-stage T cell development, possibly through the selective translational control of specific targets.

### Result 2. Conditional deletion of *Eif4g2* specifically impairs SP thymocyte development

**Figure 2:**
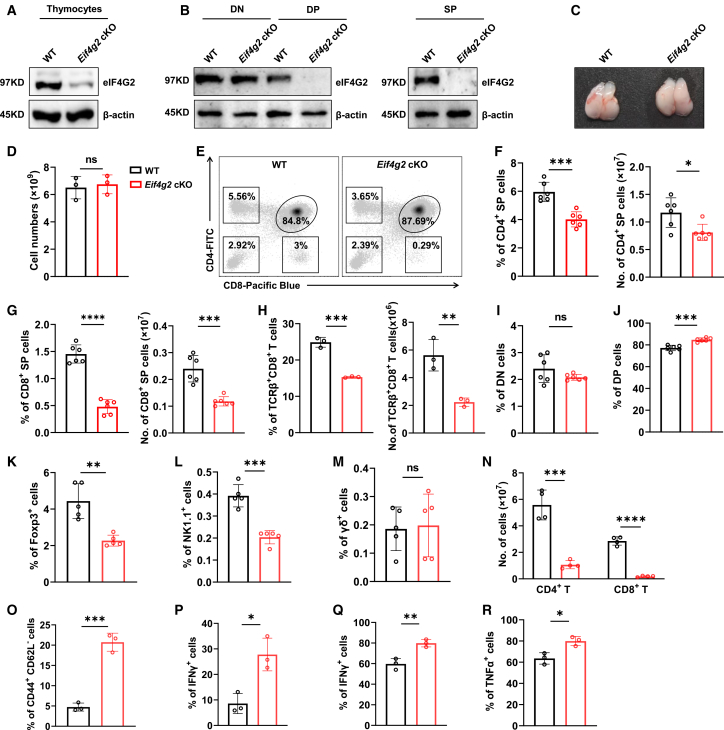
Conditional deletion of *Eif4g2* specifically impairs SP thymocyte development (A and B) Validation of eIF4G2 protein deletion by western blot. (A) Total thymocytes. (B) Lysates from sorted thymocyte subsets: double-negative (DN), double-positive (DP), and single-positive (SP) cells. (C) Representative images of thymus from WT and *Eif4g2* cKO mice. (D) Total thymocytes numbers from WT and *Eif4g2* cKO mice (*n* = 3 per group, ns *p* > 0.05). (E) Flow cytometric analysis of thymocyte populations. (F–H) Relative frequencies and absolute numbers of (F) CD4 SP, (G) CD8 SP, and (H) TCRβ^+^CD8^+^ subsets (n = 3–6 mice per group, presented as mean ± SEM. ∗*p* < 0.05, ∗∗*p* < 0.01, ∗∗∗*p* < 0.001, ∗∗∗∗*p* < 0.001). (I–M) Frequencies of (I) DN, (J) DP cells within total thymocytes, and (K) Foxp3^+^ cell within CD4^+^ T cells (n = 5–6 mice per group, presented as mean ± SEM, ns *p* > 0.05, ∗∗*p* < 0.01, ∗∗∗*p* < 0.001); (L and M) Analysis of innate-like T cells in the thymus. (L) Frequencies of NK1.1^+^ T cells and (M) γδ T cells (*n* = 5 mice per group, presented as mean ± SEM. ns *p* > 0.05, ∗∗∗*p* < 0.001). (N–P) Evaluation of peripheral T cells *in situ*. (N) Absolute numbers of splenic T cells, (O) frequencies of CD44^+^CD62L^−^ cells and (P) IFNγ^+^ cells (*n* = 3 mice per group, presented as mean ± SEM. ∗*p* < 0.05, ∗∗∗*p* < 0.001, ∗∗∗∗*p* < 0.0001). (Q and R) Cytokine production upon stimulation. Frequencies of (Q) IFNγ^+^ and (R) TNFα^+^ cells among peripheral naive T cells following *ex vivo* anti-CD3/CD28 stimulation (*n* = 3 mice per group, bar graphs show mean ± SEM. ∗*p* < 0.05, ∗∗*p* < 0.01). Data are representative of at least two independent experiments unpaired Students’*t* test was used to perform the statistical analysis. See also Figure S2.

To clarify the role of eIF4G2 in T cell development, we generated T cell-specific *Eif4g2* knock out mice (hereafter referred to as *Eif4g2* cKO) by crossing *Eif4g2*^flox/flox^ mice with the *Cd4*-*Cre* transgenic mice. *Eif4g2*^flox/flox^ mice or *Cd4*-*Cre* transgenic mice (hereafter referred to as WT) were used as control group (Figures S2A and S2B). Immunoblot analysis showed a marked reduction of eIF4G2 protein in total thymocytes, with residual signal attributable to *Cd4*-*Cre*-negative stromal and DN cells (Figure 2A). This depletion was near complete in sorted DP and SP thymocytes but not in DN cells, confirming efficient and stage-specific deletion (Figure 2B).

Given that eIF4G1 is a structural homolog of eIF4G2 and a core component of the canonical translation machinery,^14^ we sought to rule out the possibility that eIF4G1 upregulation might functionally compensate for the loss of eIF4G2 and thereby confound the interpretation of our knockout phenotypes. We assessed eIF4G1 expression in purified DP and SP thymocytes from WT and *Eif4g2* cKO mice. Western blot analysis revealed that eIF4G1 protein levels remained unchanged in *Eif4g2* deficient cells compared to WT group (Figure S2C). This result indicates that a compensatory increase in eIF4G1 expression does not occur upon *Eif4g2* ablation, supporting the conclusion that the observed developmental defects are attributable to the loss of eIF4G2-specific function.

Next, we assessed the impact of *Eif4g2* depletion on T cell development. Compared to WT group, *Eif4g2* cKO mice exhibited normal thymus size and morphology (Figure 2C). Additionally, the total cellularity of the thymus remained unaltered in *Eif4g2* cKO mice (Figure 2D). However, flow cytometry analysis revealed a pronounced reduction in both frequency and absolute number of SP thymocytes from *Eif4g2* cKO mice (Figures 2E–2G). This defect was most pronounced in the CD8 SP compartment and included mature CD8 SP thymocytes (Figures 2G, 2H, and S2D). In contrast, the percentage of DN cells showed no significant change, while the ratio of DP cells was modestly increased (Figures 2I and 2J). The developmental impairment extended to specific SP-derived lineages: both thymic regulatory T cells (Tregs), a subset of CD4^+^ T cells, and natural killer T (NKT) cells, whose development depends on the CD4 SP stage, exhibited reduced frequencies in *Eif4g2* cKO group (Figures 2K, 2L, and S2E), whereas γδ T cell development remained intact (Figure 2M).

In peripheral lymphoid organs, *Eif4g2* cKO mice exhibited a marked decrease in T cell numbers (Figure 2N). The remaining peripheral T cells showed an increased frequency of CD44^+^CD62L^−^ memory phenotype cells and elevated effector cytokine production (Figures 2O and 2P), consistent with lymphopenia driven homeostatic proliferation.^19^ When peripheral naive T cells were purified and activated with anti-CD3 and anti-CD28 antibody *in vitro*, *Eif4g2* depletion did not impair, even slightly enhanced effector cytokine production (Figures 2Q and 2R). Thus, it seems that the altered peripheral T cell compartment should stem from the thymic developmental defect, as evidenced by the intact effector function of *Eif4g2* deficient naive T cells. Collectively, these data indicate that eIF4G2 is dispensable for early thymocyte development but is required for establishing the mature SP thymocyte pool.

### Result 3. eIF4G2 facilitates CD8^+^ T lineage commitment after positive selection

**Figure 3:**
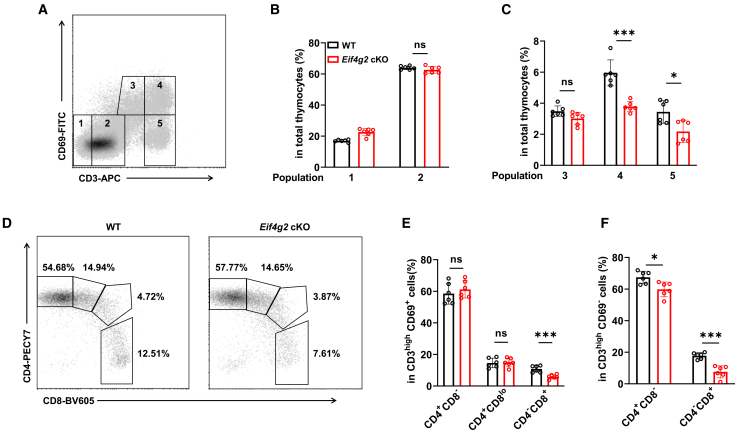
eIF4G2 facilitates CD8^+^ T lineage commitment after positive selection (A) Flow cytometry gating strategy for WT and *Eif4g2* cKO thymocytes based on CD3 and CD69 expression and representative plots for WT and *Eif4g2* cKO mice are shown. (B and C) Quantification of the thymocyte subpopulations defined in (A). Bar graphs show the frequencies of each population within total thymocytes (*n* = 6, ns *p* > 0.05, ∗∗∗*p* < 0.001, ∗*p* < 0.05). (D) Gating strategy to analyze CD4 and CD8 expression within CD3^high^CD69^+^ population. (E) Ratio of CD4^+^CD8^−^, CD4^+^CD8^lo^, or CD4^−^CD8^+^ cells in CD3^high^CD69^+^ population (*n* = 6, ns *p* > 0.5, ∗∗∗*p* < 0.001). (F) Ratio of CD4^+^CD8^−^ or CD4^−^CD8^+^ cell in CD3^high^CD69^-^ population (*n* = 6, ∗*p* < 0.5, ∗∗∗*p* < 0.001); Data are representative of at least two independent experiments. Bar graphs show mean ± SEM and unpaired Students’*t* test was used to perform the statistical analysis.

To pinpoint the developmental stage at which eIF4G2 functions, we analyzed thymocytes based on surface CD3 and CD69 expression, which delineates developmental progression^20^ (Figure 3A). In *Eif4g2* cKO mice, the frequencies of CD3^−^CD69^−^ (population 1, DN) and CD3^low^CD69^-^ populations (population 2, pre-positive selection DP) were comparable to those in WT mice. Similarly, the proportion of CD3^int^CD69^+^ cells (population 3), representing thymocytes undergoing positive selection, showed no significant alteration (Figures 3B and 3C). In contrast, the frequency of CD3^high^CD69^+^ cells (population 4) was significantly reduced in *Eif4g2* cKO group, which includes transitional cells that just completed positive selection and immature SP cells that underwent lineage commitment. Furthermore, the population of mature SP cells as CD3^high^CD69^-^ (population 5) was also markedly decreased in *Eif4g2* cKO mice (Figure 3C). These data indicate that the defect in *Eif4g2* cKO mice arises after positive selection but before full SP maturation.

To further clarify the function of eIF4G2 on immature SP cells development, we further subdivided population 4 (CD3^high^CD69^+^) into CD4^+^CD8^−^ (immature CD4 SP) cells, CD4^+^CD8^lo^ (transitional cells), CD4^+^CD8^+^ (DP), and CD4^−^CD8^+^ (immature CD8 SP) subsets (Figure 3D). Strikingly, only the immature CD8 SP fraction was markedly decreased in *Eif4g2* cKO mice, while the immature CD4 SP and transitional subsets remained intact (Figure 3E). Likewise, within the mature SP pool (population 5), the frequency of CD8 SP thymocytes was specifically and severely reduced, whereas the CD4 SP subset was only modestly affected (Figure 3F). Together, these results position the defect immediately after positive selection, specifically at the stage of immature CD8 SP cell generation, thereby explaining the severe loss of mature CD8^+^ T cells and pointing to a potential disruption of lineage-commitment signals.

To definitively map the developmental requirement for eIF4G2, we integrated the frequencies of all major thymocyte subsets we examined. This comprehensive analysis revealed a highly specific and stage-restricted defect. The comparable frequencies of DN and DP populations in *EIf4g2* cKO mice (Figures 2I and 2J), demonstrating that early T cell progenitor development, β-selection, and the initial DP pool are intact. The observed modest increase in DP frequency is consistent with a downstream developmental blockade leading to a slight accumulation of pre-selection cells. The essential defect emerged exclusively after positive selection, as evidenced by the severe and specific loss of immature CD8 SP cells within the post-selection (CD3^hi^CD69^+^) compartment (CD4^−^CD8^+^, as shown in Figure 3E) and the consequent decrease of mature CD8 SP thymocytes (Figure 2H). In contrast, the generation of CD4^+^ lineage cells were largely preserved at the immature stage (CD4^+^CD8^−^, shown in Figure 3D). Collectively, these data position the essential function of eIF4G2 not at the initial DP stage or during positive selection per se, but specifically at the execution point of CD8^+^ T cell lineage commitment immediately thereafter.

### Result 4. *Eif4g2* deletion specifically ablates the IL-7 response

**Figure 4:**
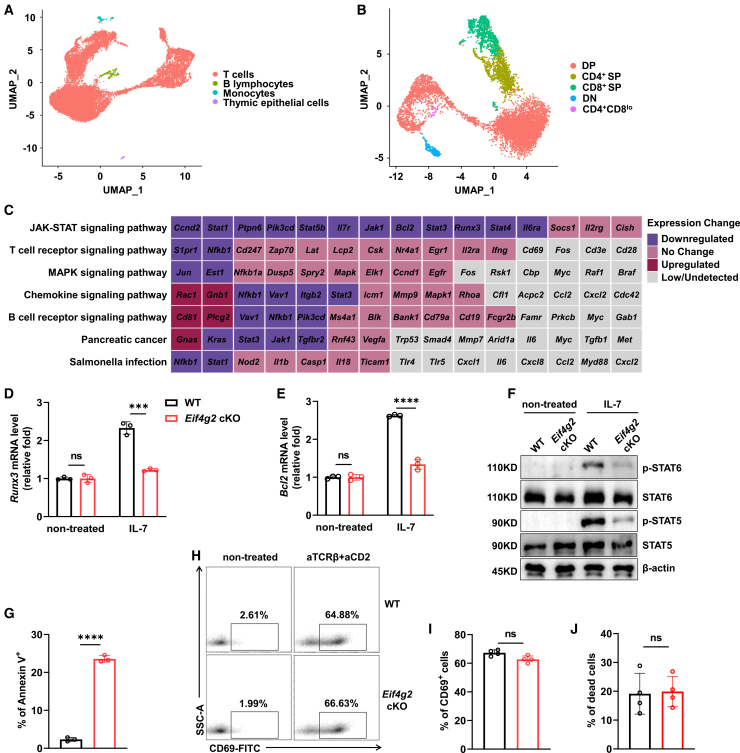
*Eif4g2* deletion specifically ablates the IL-7 response (A and B) Single-cell transcriptomic landscape of thymocytes. UMAP visualization of (A) all thymic cells and (B) the subtypes of T cells, color-coded by cell type. (C) Heatmap showing the expression pattern of 15 core signaling component genes from seven selected KEGG pathways across the cell clusters identified in (A and B). (D–F) Functional response of CD4^+^CD8^lo^ transitional cells to IL-7. Relative mRNA level of (D) *Runx3* and (E) *Bcl2* following 10 ng/ml IL-7 stimulation (*n* = 3, ∗∗∗*p* < 0.001, ∗∗∗∗*p* < 0.0001). (F) Western blot analysis of key signaling pathway activation with or without IL-7 stimulation. (G) Quantification of cell death in peripheral naive T cells under IL-7 stimulation *ex vivo* (*n* = 3, ∗∗∗∗*p* < 0.0001). (H–J) TCR signaling evaluation in DP cells under stimulation with anti-TCRβ/CD2 *ex vivo*, including (H) representative plots of CD69 expression examination, (I) frequency of CD69^+^ cells and (J) cell death level (*n* = 4, ns *p* > 0.5). Data are representative of at least two independent experiments. Bar graphs show mean ± SEM and unpaired Students’*t* test was used to perform the statistical analysis. See also Figures S3–S5.

Given that the developmental blockade in *Eif4g2* cKO mice occurred specifically at the stage of CD8^+^ lineage commitment (Figures 2 and 3), a process known to be critically dependent on cytokine signaling,^6^^,^^21^ we reasoned that the molecular defect likely resided in the dysregulation of key cytokine-responsive pathways. To test this and to identify the specific pathway affected, we performed single-cell RNA sequencing (scRNA-seq) on WT and *Eif4g2* cKO thymocytes. After quality control, we obtained 4597 WT and 4049 *Eif4g2* cKO cells. Unsupervised clustering identified major cell types within the thymus, including T cells, B lymphocytes, monocytes, and thymic epithelial cells (Figures 4A and S3A–S3D). Re-clustering of the T cell compartment further resolved developmental stages, including DN, DP, CD4^+^CD8^lo^ transitional cells, SP cells (including both CD4^+^ and CD8^+^ SP) (Figures 4B, S3E, and S3F). We focus on the CD4^+^CD8^lo^ transitional and CD8 SP populations where the developmental defect manifests.

Unbiased differential expression analysis identified 574 genes with altered expression in the cKO cells (191 upregulated, 383 downregulated). Initial differential gene set enrichment analysis (GSEA) returned a broad list of nominally significant pathways (Figure S4). However, we recognized a fundamental limitation of this approach: the presence of differentially expressed genes within a pathway annotation does not, by itself, demonstrate that the pathway’s core signaling function is compromised. Many such genes may be peripheral regulators, broadly expressed components, or shared nodes (e.g., *Nfkb1* and *Pik3cd*) whose alteration does not equate to a collapse of the pathway’s defining input-output logic. To identify which signaling pathways were dysregulated to cause the specific CD8^+^ lineage blockade, we designed a functional centric audit. We implemented a curated audit strategy designed to fulfill two objectives: first, to test the integrity of pathways critical for post-selection biology, and second, to control for pathways commonly enriched as GSEA false positives. Accordingly, we selected seven representative pathways, including the most critical ones for post-selection thymocyte biology (T cell receptor signaling, JAK-STAT signaling, and chemokine signaling), pathways that frequently appear as GSEA false positives due to shared signaling components (MAPK signaling and B cell receptor signaling), as well as the classic false positive examples that are often spuriously enriched in immune datasets (Salmonella infection and pancreatic cancer) (Figure 4C). For each pathway, we moved beyond the simple list of altered genes and instead interrogated the expression status of its canonical, non-redundant core components, and biological output. This design allowed us to differentiate between a targeted defect and global dysregulation.

This audit revealed a striking, hierarchical disparity. The JAK-STAT signaling pathway, which plays the centrol role in cytokine response, exhibited a catastrophic and coordinated collapse across its entire functional axis (Figure 4C), which validated and refined our initial postulate. This failure was comprehensive: definitive downstream effector genes controlling lineage commitment (*Runx3*), survival (*Bcl2*), and proliferation (*Ccnd2*) were suppressed, a direct consequence of the concurrent downregulation of essential signaling components, including key kinase (*Jak1*), and central transcription factors (*Stat5b*, *Stat1*). Strikingly, we observed the *Il7r* (encoding IL-7Rα) mRNA level was also decreased, which led us to hypothesize that the defect lies in IL-7 receptor signaling, the likely root cause of the developmental block in *Eif4g2* deficient T cells. In contrast, the decisive functional output of other critical pathways remained operational, despite some alterations in individual gene expression (Figure 4C). For TCR signaling, the immediate-early genes *Nr4a1*, *Egr1* remained unchanged, and the activation marker *Il2ra* was normally induced. For MAPK signaling, despite some sporadic changes (*Jun*), functional readouts were preserved, with the expression of direct downtream induction genes (*Dusp5*, *Spry2*) and the cell cycle effector *Ccnd1* remained unaltered. Chemokine signaling output was also preserved, with stable expression of effectors *Icam1* and *Mmp9*. Notably, the reduction of the pleiotropic signaling node NF-κB1 (*Nfkb1*), observed in both TCR signaling and chemokine signaling contexts, did not translate into a broad failure of NF-κB-responsive transcription, as its canonical downstream targets (e.g., *Nfkb1a*, *Icam1*, and *Mmp9*) remained unaffected. This demonstrates that the alteration of this shared component did not lead to a broad failure in the functional output of the pathways it inhabits. Critically, this pattern of isolated, non-propagating alterations, encompassing *Nfkb1* and other sporadic changes, was also the hallmark of the control pathways (B cell receptor signaling and Salmonella infection), confirming them as likely analytical artifacts. Thus, our analysis pinpointed a specific and catastrophic failure in the cytokine-responsive JAK-STAT pathway (largely possibly, the IL-7R signaling), while exonerating other core signaling axes, as the definitive molecular lesion responsible for the blocked CD8^+^ lineage commitment.

To test this hypothesis, we purified CD4^+^CD8^lo^ transitional cells from *Eif4g2* cKO thymocytes and stimulated with recombinant IL-7 *in vitro*, followed by an assessment of downstream signaling activation (Figure S5). In *Eif4g2* cKO cells, the induction of key IL-7 targeting genes, such as *Runx3* and *Bcl2*^3^ was severely blunted at the mRNA level (Figures 4D and 4E). At the protein level, IL-7 triggered phosphorylation of STAT5 and STAT6 was dramatically reduced, whereas total STAT protein amounts were unchanged (Figure 4F). These data suggest that eIF4G2 is required for efficient IL-7R signaling in thymocytes undergoing lineage commitment.

Also, given the known role of IL-7 signal in maintaining naive T cell survival, we asked whether eIF4G2 is also necessary for this peripheral homeostasis. Naive T cells purified from the spleen of *Eif4g2* cKO mice were cultured in the presence of recombinant IL-7. *Eif4g2* deficient cells exhibited a dramatic higher frequency of apoptosis compared to WT cells (Figure 4G). This demonstrates that the requirement for eIF4G2 in sustaining effective IL-7R signaling extends to mature T cells and may contribute to the reduction in mature CD4 SP thymocytes observed earlier (Figure 3F).

To determine the specificity of this signaling defect, we examined TCR responsiveness, which is essential for positive selection and CD4^+^ lineage commitment. DP thymocytes from WT and *Eif4g2* cKO mice were stimulated with antibodies against TCRβ and CD2 to mimic physiological TCR engagement. Under this stimulation, *Eif4g2* deficient DP cells upregulated the early activation marker CD69 to a level comparable to that of WT cells (Figures 4H and 4I). We further assessed another key functional outcome of TCR signaling, the support of cell survival. No significant difference in the rate of cell death was observed between WT and *Eif4g2* cKO group (Figure 4J).

Collectively, these data establish that the essential function of eIF4G2 is specific to the IL-7R signaling axis. Its loss ablates IL-7 responsiveness but spares the core functionality of other key developmental pathways, including TCR signaling.

### Result 5. eIF4G2 specifically sustains surface expression of the IL-7 receptor

**Figure 5:**
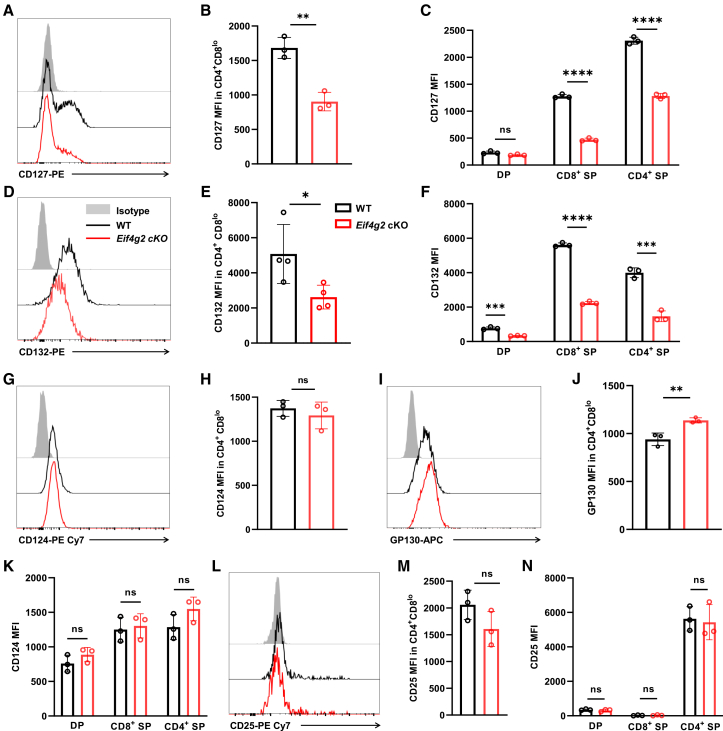
eIF4G2 specifically sustains surface expression of the IL-7 receptor (A–C) Expression of IL-7Rα (CD127). (A) Representative histogram and (B) quantification of CD127 median fluorescence intensity (MFI) on CD4^+^CD8^lo^ transitional cells (*n* = 3, ∗∗*p* < 0.01). (C) MFI of CD127 on DP, CD4 SP, and CD8 SP thymocytes (*n* = 3, ns *p* > 0.05, ∗∗∗∗*p* < 0.0001). (D–F) Expression of the common γc (CD132). (D) Representative histogram and (E) quantification of CD132 MFI on CD4^+^CD8^lo^ transitional cells (*n* = 4, ∗*p* < 0.05). ppp(F) MFI of CD132 on DP, CD4 SP, and CD8 SP thymocytes (*n* = 3, ∗∗∗*p* < 0.001, ∗∗∗∗*p* < 0.0001). (G and H) Expression of IL-4Rα (CD124) expression detection on CD4^+^CD8^lo^ transitional cells. (G) Representative histogram and (H) quantification of CD124 MFI on CD4^+^CD8^lo^ transitional cells (*n* = 3, ns *p* > 0.05). (I and J) Expression of GP130 on CD4^+^CD8^lo^ transitional cells. (I) Representative histogram and (J) quantification of GP130 MFI on CD4^+^CD8^lo^ transitional cells (*n* = 3, ∗∗*p* < 0.01). (K) Quantification of CD124 MFI on DP, CD4 SP and CD8 SP (*n* = 3, ns *p* > 0.05). (L–N) Expression of IL-2Rα (CD25). (L) Representative histogram and (M) quantification of CD25 MFI on CD4^+^CD8^lo^ transitional cells (*n* = 3, ns *p* > 0.05). (N) MFI of CD25 on DP, CD4 SP, and CD8 SP subsets (*n* = 3, ns *p* > 0.05). Data are representative of at least two independent experiments. Bar graphs show mean ± SEM and unpaired Students’*t* test was used to perform the statistical analysis.

Having established that eIF4G2 is required for functional IL-7 responsive signaling, we next sought to determine the underlying molecular basis. Given the central role of IL-7R in initiating this signaling cascade, we hypothesized that eIF4G2 may regulate the expression of the receptor complex itself. Consistent with the earlier observation of diminished *Il7r* mRNA in our scRNA-seq analysis, flow cytometry revealed that surface expression of IL-7Rα (CD127) was indeed significantly reduced on *Eif4g2*-deficient transitional and SP thymocytes (Figures 5A–5C). Interesting, we also observed a profound reduction of the common γc chain (CD132), which manifested across all the thymic subsets analyzed, including CD4^+^CD8^lo^ transitional cells (Figures 5D and 5E) as well as DP, CD4 SP, and CD8 SP populations (Figure 5F). The coordinated loss of both receptor subunits provides a clear mechanistic explanation for the observed IL-7R signaling failure.

We next asked whether this regulatory function extended to other cytokine receptors. Surface expression of IL-4Rα (CD124) and GP130 was not reduced in *Eif4g2* cKO transitional cells, and even modestly elevated level of GP130 was observed (Figures 5G–5J). IL-4Rα expression was also unaltered across other detected subsets (Figure 5K). We further assessed the IL-2 receptor, another γc-dependent complex crucial for Treg cell development. As expected, IL-2Rα expression was minimal in transitional, DP, and CD8 SP thymocytes. In CD4 SP cells, where IL-2Rα is highly expressed, its level was unchanged by *Eif4*g*2* deletion (Figures 5L–5N). Collectively, these data demonstrate that the profound reduction in surface γc is not accompanied by a general downregulation of other cytokine receptor subunits. The observed thymic Treg reduction likely stems not from a loss of IL-2Rα, but from the specific decrease in the shared, essential γc subunit.

Together, these data establish that eIF4G2 is specifically required to maintain surface expression of the intact IL-7R complex across post-selection thymocyte subsets, without broadly impairing other cytokine receptor pathways.

### Result 6. eIF4G2 post-transcriptionally sustains γc expression via its mRNA UTRs

**Figure 6:**
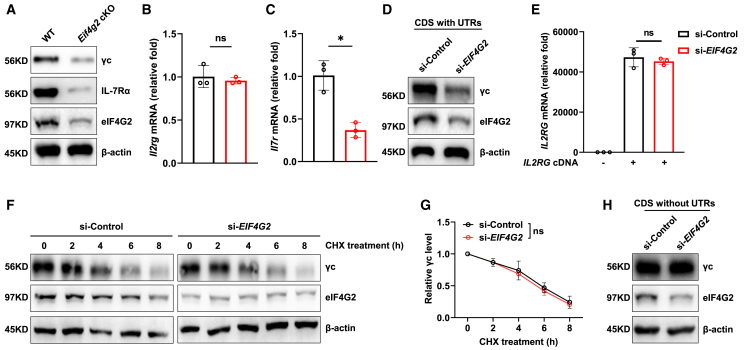
eIF4G2 post-transcriptionally sustains γc expression via its mRNA UTRs (A–C) Analysis in primary CD4^+^CD8^lo^ transitional thymocytes. (A) Western blot analysis of γc and IL-7Rα protein levels. (B and C) Quantitative RT-PCR analysis of *Il2rg* (B) and *Il7r* (C) mRNA levels (*n* = 3, ns *p* > 0.05, ∗*p* < 0.05). (D–H) Mechanistic dissection in 293T cells. (D) Western blot of γc protein in control and *EIF4G2* knockdown 293T cells transfected with an *IL2RG* coding sequence construct containing its native 5′ and 3′ UTRs. (E) Corresponding *IL2RG* mRNA levels measured by RT-qPCR (*n* = 3, ns *p* > 0.05) . (F and G) Assessment of γc protein stability (*n* = 3, ns *p* > 0.05). (F) Representative western blots and (G) quantification of γc protein levels over time following cycloheximide (CHX) treatment in si-control and si-*EIF4G2* 293T cells (*n* = 3, ns *p* > 0.05). (H) Western blot of γc protein in si-control and si-*EIF4G2* 293T cells transfected with an *IL2RG* CDS construct lacking UTRs. Data are representative of at least two independent experiments. Bar graphs show mean ± SEM and unpaired Students’*t* test was used to perform the statistical analysis.

To define the mechanism by which eIF4G2 maintains IL-7 receptor levels, we first confirmed the protein loss in purified CD4^+^CD8^lo^ transitional thymocytes by immunoblotting. Consistent with the flow cytometry data, eIF4G2 was required for the expression of both γc and IL-7Rα proteins (Figure 6A). We then sought to determine whether this regulation occurred at the transcriptional or post-transcriptional level. Quantitative RT-PCR analysis revealed a striking mechanistic divergence: the mRNA level of *Il2rg* (encoding γc) was unchanged in *Eif4g2* cKO cells, confirming a post-transcriptional defect. In contrast, *Il7r* (encoding IL-7Rα) mRNA was significantly reduced (Figures 6B and 6C), thus validating and quantifying the downregulation suggested by our prior scRNA-seq analysis (Figure 4C). These data establish that eIF4G2 maintains γc expression through a post-transcriptional mechanism, while its effect on IL-7Rα is associated with, and likely mediated by, a reduction at the mRNA level.

Based on its canonical role as a translation initiation factor and our finding that *Il2rg* mRNA is unaltered, we hypothesized that eIF4G2 sustains γc expression by directly regulating its mRNA translation. To test this, we knocked down *EIF4G2* (encoding eIF4G2 in human) in 293T cells and then transfected cells with a *IL2RG* (encoding γc in human) cDNA expressing construct containing 5′- and 3′-UTR sequence (UTRs). Under these conditions, *EIF4G2* knockdown still markedly reduced ectopic γc protein expression without affecting the transfected mRNA level (Figures 6D and 6E), indicating that the defect acts at the level of translation from this exogenous transcript. This effect was not due to altered protein stability, as γc kinetics were unchanged upon cycloheximide (CHX) treatment (Figures 6F and 6G). Most definitively, when the same experiment was performed with *IL2RG* cDNA construct lacking 5′- and 3′-UTR sequence, *EIF4G2* knockdown failed to reduce γc protein expression (Figure 6H). These results demonstrate that eIF4G2 is specifically required for the efficient translation of γc mRNA in a UTRs-dependent manner.

Together, these results establish a bipartite regulatory mechanism underpinning IL-7 receptor expression: eIF4G2 orchestrates IL-7 receptor levels by sustaining the γc subunit translationally and maintaining IL-7Rα transcriptionally. Thus, eIF4G2 emerges as a critical translational checkpoint that, by safeguarding IL-7R expression, ensures the cytokine signaling fidelity necessary for CD8^+^ T cell lineage commitment.

## Discussion

This study identifies the translation initiation factor eIF4G2 as a critical and selective regulator of CD8^+^ T cell lineage commitment. We demonstrate that eIF4G2 is essential for maintaining IL-7R signaling in developing thymocytes. Its deficiency ablates IL-7-dependent gene activation, severely blocks CD8^+^ lineage commitment, and reveals a previously unrecognized translational control point in T cell fate determination.

Although transcriptomic profiling is widely used to infer protein expressions, growing evidence suggests a poor correlation between mRNA abundance and protein levels. Underscoring the critical role of translational control in determining protein composition.^22^^,^^23^ Despite this, the contribution of translation factors to lymphocyte development remains largely unexplored. Our study revealed that eIF4G2, a factor implicated in both canonical and selective translation, is essential for a specific stage of T cell development, highlighting that post-transcriptional regulation is not merely permissive but instructive in immune cell differentiation.

The γc cytokines, particularly IL-7, are known to be the most critical factor in CD8^+^ T cell lineage commitment.^21^^,^^24^^,^^25^ In CD4^+^CD8^lo^ transitional thymocytes, effective IL-7 signaling activates STAT5 and STAT6, driving the expression of lineage-defining transcription factors such as Runx3.^3^ How this pathway is quantitatively tuned to ensure faithful lineage decisions remains unclear. Here, building on our integrated analysis combining single-cell transcriptomics with functional validation, we defined eIF4G2 as a translational rheostat that sets the threshold for IL-7 responsiveness by controlling IL-7R complex availability. The specificity of this regulation is striking, as no similar effect on proximal TCR signaling or other cytokine receptors, including IL-4Rα and GP130 (Figures 5G–5K), indicating the selective safeguards for IL-7R axis.

Mechanistically, eIF4G2 maintains γc expression post-transcriptionally. The unchanged *Il2rg* mRNA levels, unaltered protein stability, and most decisively, the loss of regulation upon removal of γc mRNA UTRs collectively demonstrate that eIF4G2 facilitates γc synthesis via a UTR-dependent translational mechanism (Figure 6H). This finding provides a molecular explanation for the dynamic regulation of γc protein levels observed in thymocytes and challenges the notion of its constitutive expression.^21^^,^^26^^,^^27^

Our data reveal that in eIF4G2 deficient thymocytes, impaired IL-7 signaling is accompanied by reduced *Il7r* transcription. This positive correlation contrasts with the negative feedback mechanism described in activated peripheral T cells, where IL-7 suppresses its own receptor expression.^28^ Instead, it aligns with scenarios observed in other contexts, such as germinal center B cells, where IL-7 can induce *Il7r* expression.^29^ This suggests that the relationship between IL-7 signaling and IL-7Rα expression is context-dependent, and during thymic CD8^+^ lineage commitment, a positive, reinforcing loop may be operational.

In addition to the profound block in CD8^+^ lineage commitment, *Eif4g2* deficiency resulted in milder but significant reduction in mature CD4 SP thymocytes. Unlike the CD8^+^ lineage, whose commitment is strictly dependent on a strong IL-7 signal, CD4 SP was primarily instructed by TCR-mediated signals.^30^ However, their post-selection maturation and intrathymic survival are critically dependent on sustained IL-7R signaling. Thus, the reduction in mature CD4 SP cells in the *Eif4g2* deficient thymus is most parsimoniously explained as a secondary survival defect, stemming from the diminished IL-7R availability. This interpretation is consistent with the unaltered frequency of immature CD4 SP cells (Figure 3E), which represent the stage immediately after CD4^+^ lineage commitment.

The reduction in γc expression resulting from *Eif4g2* deletion extends its potential impact beyond the IL-7 pathway to other γc-dependent cytokines,^5^ such as IL-2 and IL-15, which are critical for the development of Treg and NKT cells. Although surface assembly of the IL-2 receptor remained intact, diminished γc availability may lower signaling efficiency, offering a plausible mechanism for the observed reductions in thymic Treg and NKT cells beyond a general block at the SP stage. This is supported by our findings that γc expression is significantly reduced on CD4 SP thymocytes (the direct precursors of Tregs), while the expression of the IL-2 receptor α chain remains unchanged (Figure 5N). The resultant compromise in IL-2 receptor signaling competence provides a specific and parsimonious explanation for the impaired development of CD25^+^Foxp3^+^ Tregs in *Eif4g2* deficient mice. Thus, eIF4G2 emerges not merely as a facilitator of IL-7 signaling but as a broader translational coordinator of γc cytokine responses, fine-tuning multiple lineage outcomes during thymocyte development.

In conclusion, we define a translational control layer in T cell development, wherein eIF4G2 selectively licenses CD8^+^ lineage commitment by ensuring the expression of key cytokine receptor subunits. This work shifts the paradigm from viewing translation as a generic housekeeping process to recognizing factor driven, mRNA-specific translation as a powerful mechanism for precise spatial and temporal control in cell fate decisions.

### Limitations of the study

While our study establishes that eIF4G2 sustains IL-7R expression via bipartite control of γc translation and IL-7Rα transcription, several mechanistic and biological questions remain. First, we did not globally profile the translatome in the relevant thymic subsets, therefore, the full spectrum of mRNAs whose translation depends on eIF4G2 remains undefined. Second, the precise step in translation initiation regulated by eIF4G2 on the γc mRNA is unknown. Third, the reduction in IL-7Rα mRNA could be an indirect consequence of diminished IL-7R signaling or may involve more direct transcriptional regulation. Forth, although our focused audit pinpointed the JAK-STAT axis as the definitive lesion, the numerous other transcriptional alterations observed, particularly in metabolic and adhesion pathways, may represent secondary adaptations or unmask additional, subtler functions of eIF4G2 that our current analysis did not capture. Finally, our work centers on a late developmental checkpoint. Whether eIF4G2 governs earlier thymic stages or peripheral T cell homeostasis through similar or distinct mechanisms awaits future investigation.

## Resource availability

### Lead contact

Requests for further information and resources should be directed to and will be fulfilled by the lead contact, Xueting Lang (lxting88@ihm.ac.cn).

### Materials availability

Materials listed in this manuscript are available from the lead contact upon reasonable request.

### Data and code availability

• Data: The single-cell RNA sequencing data have been deposited in the NCBI Gene Expression Omnibus (GEO) database, with the corresponding accession numbers GEO: GSE317990, are publicly available. Custom R scripts for RNA analysis used in this paper are also available upon request. All original data have been deposited at Mendeley Data: https://data.mendeley.com/datasets/8fzxckf7ym/2 and are publicly available as of the date of publication.
• Code: This paper does not report original code.
• Other items: Additional data required for reanalysis of this study can be obtained from the corresponding author upon reasonable request.

## Acknowledgments

We thank Dr. Yuanwei Zhang for his expert technical assistance with single-cell RNA sequencing data analysis. We also acknowledge the Core facility of Institute of Health and Medicine, Hefei Comprehensive National Science Center for animal care and technical support. This work was supported in part by the 10.13039/501100001809National Natural Science Foundation of China to X.L. (reference number 82371854); the Anhui Key Research and Development Plan (reference number 2023z04020011); the start-up funds from the Institute of Health and Medicine, Hefei Comprehensive National Science Center (reference number 2022KYQD008).

## Author contributions

Conceptualization, funding acquisition, project administration, supervision, writing review and editing: W.X., T.J., and X.L.; investigation, formal analysis, validation, visualization, writing original draft: J.C. and X.Z.; investigation, methodology Y.Y. and L.S.; data curation, writing review and editing: Y.L. and L.J.

## Declaration of interests

The authors declare no competing interests.

## Declaration of generative AI and AI-assisted technologies in the writing process

During the preparation of this work, the authors used DeepSeek Chat solely for the purpose of improving the readability and polish the English language of the manuscript. The tool was not used to generate or analyze any research data or scientific conclusions. After using this tool, the authors reviewed and edited the content as needed and take full responsibility for the content of the publication.

## STAR★Methods

### Key resources table

**Table:** 

REAGENT or RESOURCE	SOURCE	IDENTIFIER
**Antibodies**		

Mouse Monoclonal Anti-beta-Actin (8H10D10)	Cell Signaling Technology	Cat# 3700S; RRID: AB_2242334
Rabbit Monoclonal Anti-eIF4G2/p97 (D1A10)	Cell Signaling Technology	Cat# 3468S; RRID: AB_2261993
Rabbit Monoclonal Anti-Phospho-Stat5 (Tyr694) (D47E7)	Cell Signaling Technology	Cat# 4322T; RRID: AB_10544692
Rabbit Monoclonal Anti-Phospho-Stat6 (Tyr641) (D8S9Y)	Cell Signaling Technology	Cat# 56554S; RRID: AB_2799514
Rabbit Polyclonal anti-IL-7Rα/CD127	Proteintech	Cat# 17626-1-AP; RRID: AB_2126105
Rabbit Polyclonal anti-IL-2RG	Proteintech	Cat# 11409-1-AP; RRID: AB_2264645
Rabbit Polyclonal anti-STAT5A/B	Proteintech	Cat# 13179-1-AP; RRID: AB_2196760
Rabbit Polyclonal anti-STAT6	Proteintech	Cat# 51073-1-AP; RRID: AB_2197244
Rabbit Polyclonal anti-eIF4G1	Proteintech	Cat# 15704-1-AP; RRID: AB_2261979
HRP-conjugated Goat Anti-Mouse IgG (H+L)	Proteintech	Cat# SA00001-1; RRID: AB_2722565
HRP-conjugated Donkey Anti-Rabbit IgG (H+L)	Proteintech	Cat# SA00001-9; RRID: AB_2890888
Rat Monoclonal Anti-CD8α (53-6.7), FITC	Thermo Fisher Scientific	Cat# 11-0081-82; RRID: AB_464915
Rat Monoclonal Anti-CD4 (GK1.5), FITC	Thermo Fisher Scientific	Cat# 11-0041-85; RRID: AB_464893
Armenian hamster Monoclonal Anti-CD69 (H1.2F3), FITC	Thermo Fisher Scientific	Cat# 11-0691-82; RRID: AB_465119
Rat Monoclonal Anti-TNF alpha (MP6-XT22), APC	Thermo Fisher Scientific	Cat# 17-7321-82; RRID: AB_469508
Rat Monoclonal Anti-FOXP3 (FJK-16s), FITC	Thermo Fisher Scientific	Cat# 11-5773-82; RRID: AB_465243
Rat Monoclonal Anti-CD127 (A7R34), PE	Thermo Fisher Scientific	Cat# 12-1271-81; RRID: AB_465843
Rat Monoclonal Anti-CD3 (17A2), APC	Thermo Fisher Scientific	Cat# 17-0032-82; RRID: AB_10597589
Mouse Monoclonal Anti-CD45.2 (104), PE-Cyanine7	Thermo Fisher Scientific	Cat# 25-0454-82; RRID: AB_2573350
Rat Monoclonal Anti-IFN gamma (XMG1.2), PerCP-Cyanine5.5	Thermo Fisher Scientific	Cat# 45-7311-82; RRID: AB_1107020
Armenian hamster Monoclonal Anti-TCR beta (H57-597), FITC	Thermo Fisher Scientific	Cat# 11-5961-82; RRID: AB_465323
Rat Monoclonal Anti-CD25 (PC61.5), PE-Cyanine7	Thermo Fisher Scientific	Cat# 25-0251-82; RRID: AB_469608
Mouse Monoclonal Anti-CD4 (RM4-5), PE-Cyanine7	Thermo Fisher Scientific	Cat# 25-0042-82; RRID: AB_469578
Rat Monoclonal Anti-CD62L (MEL-14), PE	Thermo Fisher Scientific	Cat# 12-0621-81; RRID: AB_465720
Rat Monoclonal Anti-CD44, PerCP	BioLegend	Cat# 103035; RRID: AB_10639933
Armenian hamster Monoclonal Anti-TCR γ/δ, APC	BioLegend	Cat# 118115; RRID: AB_1731824
Rat Monoclonal Anti-CD132 (common γ chain), PE	BioLegend	Cat# 132305; RRID: AB_2123703
Rat Monoclonal Anti-CD130 (gp130), APC	BioLegend	Cat# 149405; RRID: AB_2927933
Rat Monoclonal Anti-CD124 (IL-4Rα), PE/Cyanine7	BioLegend	Cat# 144805; RRID: AB_2565598
Mouse Monoclonal Anti-NK-1.1, PE/Cyanine7	BioLegend	Cat# 108714; RRID: AB_389364
Rat Monoclonal Anti-CD8α, Brilliant Violet 421	BioLegend	Cat# 100737; RRID: AB_10897101
Mouse Monoclonal Anti-CD45.2, PE	BioLegend	Cat# 109807; RRID: AB_313444
Armenian hamster Monoclonal Ultra-LEAF™ Purified Anti-TCR β chain	BioLegend	Cat# 109253; RRID: AB_2813970
Rat Monoclonal Ultra-LEAF™ Purified Anti-CD2	BioLegend	Cat# 100118; RRID: AB_2832256
Pharmingen™ Purified NA/LE Hamster Anti-Mouse CD28	BD Biosciences	Cat# 553294; RRID: AB_394763
Pharmingen™ Purified NA/LE Hamster Anti-Mouse CD3e	BD Biosciences	Cat# 553057; RRID: AB_394590

**Chemicals, peptides, and recombinant proteins**		

Mouse IL-2 Recombinant Protein	PeproTech	Cat# 212-12
Mouse IL-7 Recombinant Protein	PeproTech	Cat# 217-17
2-Mercaptoethanol	Gibco	Cat# 21985023
RPMl Medium 1640 basic (1X)	Gibco	Cat# 8123426
Fetal Bovine Serum (FBS)	Gibco	Cat# 10091148
Phosphate Buffered Saline (PBS) pH 7.4 (1X)	Gibco	Cat# 10010023
Opti-MEM™ I Reduced Serum Medium	Gibco	Cat# 11058021
Penicillin-Streptomycin Solution, 100X	Beyotime	Cat# C0222
DMEM (High Glucose) Medium	Biochannel	Cat# BC-M-005
Annexin V, APC	BioLegend	Cat# 640920
TRIzol Reagent	Invitrogen	Cat# AM9738
Trypan Blue solution	Sigma	Cat# 72-57-1

**Critical commercial assays**		

Lipofectamine™ RNAiMAX	Invitrogen	Cat# 13778075
Lipofectamine™ 2000	Invitrogen	Cat# 11668019
LIVE/DEAD™ Fixable Yellow Dead Cell Stain Kit	Invitrogen	Cat# L34968
RevertAid RT Kit	Thermo Fisher Scientific	Cat# K1691
2X Universal SYBR Green Fast qPCR Mix	Abclonal	Cat# RK21203
Cell Stimulation Blend (Plus Protein Transport Inhibitor) (500X)	eBioscience	Cat# 00-4975-93
Foxp3/Transcription Factor Staining Buffer Set	eBioscience	Cat# 00-5523-00
EasySep™ Mouse Pan-Naïve T Cell Isolation Kit	STEMCELL	Cat# 19848RF
DNBelab C Series High-throughput Single-cell RNA Library Preparation Set V3.0 (TaiM 4)-16RXNS/SET	MGI Tech	Cat# 940-001818-00
Tanon™ ECL chemiluminescent substrates	Tanon	Cat# 1805001
123count eBeads™ Counting Beads	Invitrogen	Cat# 01-1234-42

**Deposited data**		

Raw and analyzed data	Jingchang Ma et al.	GEO: GSE194270
Raw data for scRNA-seq	this paper	GEO: GSE317990
Data and statistical analysis	this paper	Mendeley: access link https://doi.org/10.17632/8fzxckf7ym.1.

**Experimental models: Cell lines**		

293T	ATCC	ATCC CRL-3216

**Experimental models: Organisms/strains**		

Mouse: C57BL/6JGpt-Eif4g2em1Cflox/Gpt	GemPharmatech	Strain# T008923; RRID: IMSR_GPT: T008923
Mouse: B6.Cg-Tg(Cd4-cre)1Cwi/BfluJ	The Jackson Laboratory	Strain# 022071; RRID: IMSR_JAX: 022071

**Oligonucleotides**		

siRNA sequence for Human *EIF4G2* 1#: GCAGUUAGCUAAAUUACAAGA	This paper	N/A
siRNA sequence for negative control NCUUCUCCGAACGUGUCACGUdTdT	This paper	N/A
Primers for Human *EIF4G2* Forward: TGGAGAGTGCGATTGCAGAA Reverse: TAGTGCTTCGTGCAGGAATC	This paper	N/A
Primers for Human *IL2RG* Forward: GGGCTGAACACGACAATTCT Reverse: TCAGAGCTGCTGTTCCAAGT	This paper	N/A
Primers for Human *ACTB* Forward: CATGTACGTTGCTATCCAGGC Reverse: CTCCTTAATGTCACGCACGAT	This paper	N/A
Primers for mouse *Actb* Forward: GGCTGTATTCCCCTCCATCG Reverse: CCAGTTGGTAACAATGCCATGT	This paper	N/A
Primers for mouse *Eif4g2* Forward: AGTGCGATTGCAGAAGGGG Reverse: GTGCTTCGTGCAGGAATCCA	This paper	N/A
Primers for mouse *Eif4g1* Forward: AAGCGACACAAATGAACACG Reverse: CCCCTGTCCAGGGATATAGT	This paper	N/A
Primers for mouse *Runx3* Forward: GACTCCTTCCCCAACTATACACC Reverse: CGCTGTTCTCGCCCATCT	This paper	N/A
Primers for mouse *Bcl2* Forward: ATGCCTTTGTGGAACTATATGGC Reverse: GGTATGCACCCAGAGTGATGC	This paper	N/A
Primers for mouse *Il7r* Forward: GCGGACGATCACTCCTTCTG Reverse: AGCCCCACATATTTGAAATTCCA	This paper	N/A
Primers for mouse *Il2rg* Forward: CTCAGGCAACCAACCTCAC Reverse: GCTGGACAACAAAT GTCTGGTAG	This paper	N/A

**Recombinant DNA**		

Plasmid: pCMV-*IL2RG* CDS	This paper	N/A
Plasmid: pCMV-*IL2RG* CDS with 5′ and 3′ UTRs	This paper	N/A
Plasmid: pCMV-Empty	This paper	N/A

**Software and algorithms**		

ImageJ	NIH	https://imagej.net
Prism 9	Graphpad	RRID: SCR_002798
RStudio	RStudio (v 4.0.5)	https://posit.co/download/rstudio-desktop/
FlowJo	BD Biosciences	https://www.flowjo.com/solutions/flowjo
Seurat	Seurat package (v4.3.0)	https://github.com/satijalab/seurat
clusterProfiler	clusterProfiler package (v4.2.2)	https://github.com/YuLab-SMU/clusterProfiler
ggplot2	ggplot2 package (v3.3.5)	https://ggplot2.tidyverse.org/
ComplexHeatmap	ComplexHeatmap package (v2.10.0)	https://github.com/jokergoo/ComplexHeatmap

### Experimental model and participant details

#### Animals

*Eif4g2*^flox/flox^ mice (Strain NO. T008923) were purchased from GemPharmatech and *Cd4*-*Cre* mice were obtained from Jackson Laboratary (Strain NO. 022071). To generate *Eif4g2*^flox/flox^ *Cd4*-*Cre* (*Eif4g2* cKO) mice, *Eif4g2*^flox/flox^ mice were crossed with *Cd4*-*Cre* mice, and the mouse line was maintained on a C57BL/6 background. Both male and female mice were used in this study, ranging from 6 to 12 weeks of age. No significant sex-specific differences were observed in the analyzed phenotypes; therefore, data from both sexes were pooled for analysis unless otherwise indicated. All mice were maintained under SPF housing with a maximum of five mice per cage. The care and breeding of mice and all animal experiments were conducted in accrordance with the guidelines approved by the Institutional Animal Care and Use Committee (IACUC) of the Institute of Health and Medicine, Hefei Comprehensive National Science Center.

#### Cell lines

293T cells were purchased from ATCC and cultured in DMEM medium. The cell line was authenticated by the supplier using short tandem repeat (STR) profiling and was used within six months of resuscitation. All cells were cultured in the indicated medium supplemented with 10% fetal bovine serum (FBS, Gibco) and 1% penicillin-streptomycin at 37°C with 5% CO2. Cells were confirmed to be mycoplasma free by PCR.

#### Primary cell culture

Primary lymphocytes from thymus and spleen were cultured in RPMI 1640 medium containing 10% FBS, 1% penicillin-streptomycin, and 50 μM β-mercaptoethanol. Specific subsets were subsequently isolated and subjected to cytokine stimulation, survival, or activation detection under specified for each assay.

### Method details

#### Western blot

Protein was extracted from the cells with RIPA buffer (Beyotime) supplemented with 100× protease inhibitor (Thermo Fisher Scientific). Proteins were separated by SDS-polyacrylamide gel electrophoresis (SDS-PAGE) and transferred onto polyvinylidene difluoride (PVDF) membranes (Millipore). Membranes were blocked with 5% non-fat milk or bovine serum albumin (BSA) in Tris-buffered saline containing 0.1% Tween-20 (TBST) for 1 hour at room temperature and then incubated overnight at 4 °C with the following primary antibodies diluted in blocking buffer: eIF4G2 (CST, 3468S), β-actin (CST, 3700S), eIF4G1 (Proteintech, 15704-1-AP), IL-7Rα (Proteintech, 17626-1-AP), γc (Proteintech, 11409-1-AP), STAT5 (Proteintech, 13179-1-AP), phospho STAT5 (CST, 4322T), STAT6 (Proteintech, 51073-1-AP), mouse phospho STAT6 (CST, 56554S). After washing with TBST, membranes were incubated with horseradish peroxidase (HRP)-conjugated goat anti-mouse or anti-rabbit secondary antibody (Proteintech, SA00001-1; SA00001-9) for 1 hour at room temperature. Protein bands were visualized using an enhanced chemiluminescence (ECL) substrate (Tanon, 1805001) and imaged with a chemiluminescence detection system (Tanon). Band intensities were quantified using ImageJ software (National Institutes of Health), normalized to β-actin or total protein loading controls, and presented as relative expression levels.

#### Flow cytometry analysis

Single-cell suspensions from mouse thymus and purified cell samples were prepared by filtering through a 70 μm strainer. For extracellular staining, samples were resuspended in fluorescence-activated cell sorting (FACS) buffer (PBS containing 2% FBS and 2 mM EDTA) with fluorochrome-conjugated anti-mouse antibodies and incubated at 4 °C for 30 minutes. The stained samples were then washed with FACS buffer. For intracellular staining, samples were fixed and permeabilized with 1× FoxP3 Fixation/Perm buffer (Thermo Fisher Scientific) for 1 hour at 4 °C, washed with 1× permeabilization buffer, and then stained with specific antibodies in permeabilization buffer for 30 minutes at 4 °C. For absolute cell number analysis, 123count eBeads™ counting beads (Thermo Fisher Scientific) were added to each sample prior to acquisition. Beads were vortexed thoroughly for 15–30 seconds to ensure a homogeneous suspension, and 100 μL of beads were added to a minimum sample volume of 300 μL. The exact number of cells per sample was calculated based on the known bead concentration and the acquired bead-to-cell event ratio. All samples were analyzed on a BD LSRFortessa, and data were processed using FlowJo software.

#### Cell purification and stimulation

CD4^+^CD8^lo^ transitional cells or DP cells were sorted from total thymic cell suspensions by CytoFLEX SRT cell sorter (Beckman Coulter). The targeted populations were identified and gated based on the expression of CD4 and CD8. For subsequenct stimulation assays, transitional cells were cultured in RPMI 1640 medium containing 10% FBS and β-Mercaptoethanol, simultaneously, stimulated with IL-7 (10 ng/mL) for 20 hours and used for subsequent analysis. DP cells were used for independent TCR stimulation assays with anti-TCRβ and anti-CD2. Naïve T cells were isolated using the EasySep™ Mouse Pan-Naïve T Cell Isolation Kit (STEMCELL Technologies) according to the manufacturer’s instructions, and viable cell counts were determined by trypan blue exlusion under light microscopte.

#### T cell activation and function assay

Single-cell suspensions were prepared from mouse spleens by grinding through a 70μm cell strainer and resuspended in 1 mL FACS buffer. Isolated naïve T cells were resuspended at a density of 2 × 10^6^ cells/mL in complete RPMI 1640 medium (supplemented with 10% fetal bovine serum, 50 μM β-mercaptoethanol, and 10 ng/mL recombinant murine IL-2). Cells were seeded into 12-well plates pre-coated with anti-CD3ε (2 μg/mL) and anti-CD28 (1 μg/mL) antibodies and cultured for 48 hours to achieve full activation. After 48 hours, activated T cells were harvested and resuspended in fresh complete medium containing a cell stimulation cocktail (plus protein transport inhibitors; 1:500 dilution, eBioscience™) to reactivate cells and inhibit cytokine secretion. Cells were incubated at 37 °C for 4–6 hours, followed by surface and intracellular staining for flow cytometric analysis of cytokine production.

#### Single-cell RNA sequencing (scRNA-seq)

Thymus samples from 5-week-old WT and *Eif4g2* cKO mice were mechanically dissociated using a 70 μm nylon sterile strainer to obtain single-cell suspensions. The single-cell suspensions were stained with trypan blue (Sigma) and viable cells were counted microscopically. Only suspensions with cell viability above 90% were used. Cells were resuspended in PBS and adjusted to a density of 1 × 10^6^ cells/mL, then loaded onto microfluidic devices for library construction and sequencing. Single-cell RNA sequencing (scRNA-seq) libraries were constructed using the DNBelab C Series Single-Cell Library Preparation Set (V3.0) (MGI, Shenzhen, China) according to the manufacturer’s instructions. Briefly, the prepared single-cell suspension was loaded onto a microfluidic chip on the DNBelab C4 portable single-cell system to co-encapsulate individual cells with barcoded magnetic beads. In each droplet, the cell was lysed, and the released mRNA molecules were captured by the beads. Following this, the emulsion was broken, and the mRNA-bound beads were collected for reverse transcription, followed by cDNA amplification and library construction. The quality of the final libraries was evaluated using an Agilent 2100 Bioanalyzer, and the concentration was quantified via Qubit dsDNA HS Assay Kit (Thermo Fisher Scientific). Qualified libraries were converted into DNA nanoballs (DNBs) and sequenced on the DNBSEQ-T7 platform (MGI) with a paired-end 100 bp (PE100) sequencing strategy.

#### scRNA-seq data analysis

##### Data sources

Single-cell RNA-seq (scRNA-seq) data from mouse thymus were derived from two independent sources: a publicly available dataset of thymus tissue from 5-week-old mice (GEO: GSE194270^18^), and a newly generated dataset in this study from thymocytes of *Eif4g2* cKO mice and their wild-type littermate controls. The raw sequencing data for the latter have been deposited in the NCBI Sequence Read Archive under BioProject (GEO: GSE317990). These data will be made publicly accessible upon manuscript acceptance.

##### Uniform data processing and quality control

Both datasets were subsequently analyzed with the Seurat R toolkit (version 4.0.5 in R v4.1.1).^31^^,^^32^ Low-quality cells were excluded based on the following quality control standards: (1) genes detected in fewer than 3 cells were excluded; (2) cells with fewer than 50 total detected genes were excluded; and (3) cells with mitochondria-expressed genes exceeding 10% were excluded. Data were normalized, scaled, and the top variable features were selected for principal component analysis (PCA).

##### Cell clustering and visualization

Cells clustering was performed using FindClusters and UMAPPlot functions in Seurat. Clusters were annotated based on marker genes identified by the FindAllMarkers function, with the following annotations and marker genes used.

For the public dataset, the following annotations and key marker genes were used: CD4 CD8 double negative T cells (DN; *Ptcra*, *Trdc*); CD4 CD8 double-positive T cells at proliferating phase (DP; *Cd4*, *Cd8α*, *Rorc*, *Mki67*, *Ccnd3*, *Rag1*, *Rag2*); CD8^+^ T (*Cd8α*, *Nkg7*, *Ms4a4b*, *Klrb1*); CD4^+^ T (*Bcl2*, *Lef1*, *Tox*, *Itm2a*); natural killer T cells (NKT) (*Cd3d*, *Klrd1*, *Klrc2*, *Nkg7*, *Xcl1*); γδT (*Cd4*-, *Cd8a*-, *Trdc*, *Trgv2*, *Tcrg-C1*, *Tcrg-C2*). For each member of the eIF family, the expression ratio in DP versus DN cells was calculated and ranked. Results were visualized using the ggplot2 R package (v3.3.5).

For the newly generated dataset, the following annotations and key marker genes were used: B lymphocytes (*Ms4a1, Cd79a, Cd79b)*; Thymic epithelial cells (*Epcam, Krt8, Cdh1, Aire, Ccl19*); Fibroblasts (*Col1a2, Col1a1*); Conventional type 1 dendritic cells (cDC1) (*Xcr1, Clec9a*); Plasmacytoid dendritic cells (pDCs) (*Siglech, Bst, Tlr7*); Neutrophils (*Lyz, Lcn2, Camp, Csf3r, Cxcr2*); Conventional type 2 dendritic cells (cDC2) (*Cd209a, Irf8*) Migratory dendritic cells (Migratory DCs) (*Ccr7, Ccl22, Ccl17, Nudt17, Cacnb3*); Cycling cells (*Mki67, Top2a, Stmn1*); Macrophages (*Mrc1, C1qc, C1qb, Cd68*); Monocytes (*Ly6c2, Ccr2, Chil3, Cx3cr1, Ifitm3*); and T cells (*Cd3d/e/g, Cd2, Trac, Trbc1*). Annotations and genes for subtypes of T cells are shown below: double negative T cells (DN; *Ptcra, Trdc*); CD4/CD8 double-positive T cells at proliferating phase (DP; *Cd4, Cd8a, Rorc, Mki67, Ccnd3*, *Cd4, Cd8a, Rag1, Rag2*); CD4^+^ SP (*Bcl2, Lef1, Tox, Itm2a*); CD8^+^ SP (*Cd8a, Nkg7, Ms4a4b, Klrb1*); CD4^+^CD8^lo^ (*Cd4-high, Cd8a-low*). To identify transcriptional changes resulting from *Eif4g2* deletion, differential gene expression analysis was conducted. Specifically, we compared cells within the CD8^+^ SP cluster and the CD4^+^CD8^lo^ clusters between WT and *Eif4g2* cKO mice using the FindMarkers function in Seurat (Wilcoxon rank-sum test). Genes with a p value < 0.05 and an absolute log2 fold-change > 0.5 were considered significantly differentially expressed. Significantly dysregulated genes were subsequently subjected to Kyoto Encyclopedia of Genes and Genomes (KEGG) pathway enrichment analysis using the clusterProfiler package (v4.2.2). Heatmaps depicting differential expression patterns and bar plots summarizing enriched pathways were generated using the ComplexHeatmap and ggplot2 packages, respectively.

#### Quantitative PCR analysis

Total RNA was extracted using TRIzol reagent (Invitrogen) followed by phenol-chloroform phase separation and isopropanol precipitation. Complementary DNA (cDNA) was synthesized from 1 μg of total RNA using the SuperScript™ III First-Strand Synthesis System (Invitrogen, K1691) with oligo(dT) primers, according to the manufacturer’s protocol. Quantitative PCR (qPCR) was performed using SYBR Green master mix (Abclonal, RK21203) on a StepOnePlus™ Real-Time PCR System (Applied Biosystems. The housekeeping gene *Actb* (β-actin) was used as an internal reference for normalization. Relative mRNA expression levels were calculated using the 2^−ΔΔCt^ method and are presented as fold change relative to the indicated control groups.

#### Cell death quantification

Cell death was assessed using the LIVE/DEAD™ Fixable Yellow Dead Cell Stain Kit (Invitrogen, L34968). Cells were resuspended in phosphate-buffered saline (PBS) and incubated with the viability dye for 15 minutes at room temperature in the dark. Unbound dye was removed by washing with PBS and analyzed by flow cytometry. To specifically assess apoptosis in peripheral naïve T cells following IL-7 stimulation, an Annexin V-based assay was performed. Cells were harvested after IL-7 treatment, washed with cold PBS, and resuspended in 1× Annexin V Binding Buffer. They were then stained with APC-conjugated Annexin V (BioLegend) and analyzed by flow cytometry.

#### Transfections of siRNA and plasmids

293T cells were seeded in 12-well plates and cultured until reaching approximately 60%-80% confluence. Cells were transfected either with a non-targeting control siRNA (si-Control) or human *EIF4G2* targeting siRNA (si-*EIF4G2*) (Sangon Biotech) using Lipofectamine RNAiMAX transfection reagent (Invitrogen). Cells were maintained in normal growth medium, with medium changes and passaging as needed, for 48–72 hours to achieve stable knockdown before proceeding to plasmid transfection. The coding sequence (CDS) of human *IL2RG* (encoding γc) was cloned—with or without its native 5′ and 3′ untranslated regions (UTRs)—into an appropriate mammalian expression vector using PCR and homologous recombination. For the overexpression assay, the si-*EIF4G2* cells and si-Control cells were transfected with 500ng of respective *IL2RG* expresson plasmid using Lipofectamine 2000 (Invitrogen). Cells were harvested 16 hours after plasmid transfection. Whole-cell lysates were prepared and subjected to western blotting to assess γc protein expression levels.

### Quantification and statistical analysis

Unless otherwise specified, statistical analyses for comparisons between two groups were performed using an unpaired, two-tailed Student’s t-test. Data are presented as mean ± SEM. A p value of < 0.05 was considered statistically significant. Sample sizes for animal and cell-based experiments were determined based on empirical observations from pilot studies and prior literature in the field, and no formal statistical method was used for sample size predetermination. All statistical tests for experimental data were conducted using GraphPad Prism software (version 9.0), and for scRNA-seq data, differential expression analysis (Wilcoxon rank-sum test) was followed by KEGG pathway enrichment analysis within defined cell clusters.

## Supplemental information

Document S1. Figures S1-S5 and Unedited WB images and independent repeats
