# Supplementary material for: Translation factor eIF4G2 directs CD8+ T cell lineage commitment by selectively enabling the IL-7 receptor response
Source: iScience. 2026 Mar 11;29(4):115313. doi: 10.1016/j.isci.2026.115313 (PMC13049607; doi:10.1016/j.isci.2026.115313)

iScience, Volume *29*

Supplemental information

Translation factor eIF4G2 directs CD8$^+$ T cell lineage commitment by selectively enabling the IL-7 receptor response

Jialong Cui, Xinhui Zhang, Yang Yang, Lidong Shan, Yang Li, Long Jiang, Wei Xie, Tengchuan Jin, and Xueting Lang

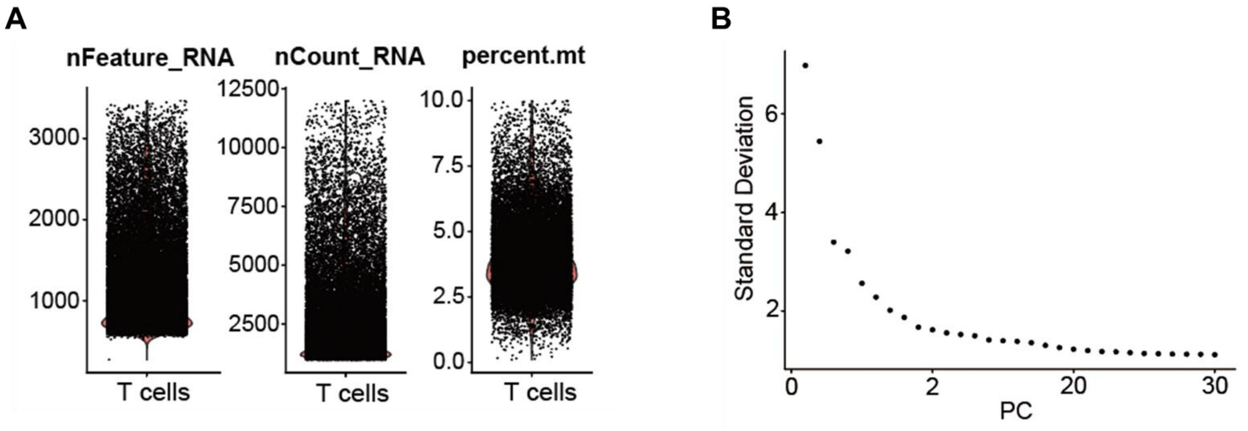

Figure S1. Quality control and initial dimensionality reduction of thymic scRNA-seq data, related to Figure 1.

(A) Violin plots showing the distribution of key quality control metrics across all cells before filtering: the number of unique genes detected per cell (nFeature_RNA), total UMI counts per cell (nCount_RNA), and percentage of mitochondrial gene expression per cell (percent.mt). Dotted lines indicate the threshold used for filtering; (B) Principal component analysis (PCA) plot of the filtered dataset. The plot displays the first 30 principal components, which were selected for downstream analysis based on an elbow plot of explained variance.

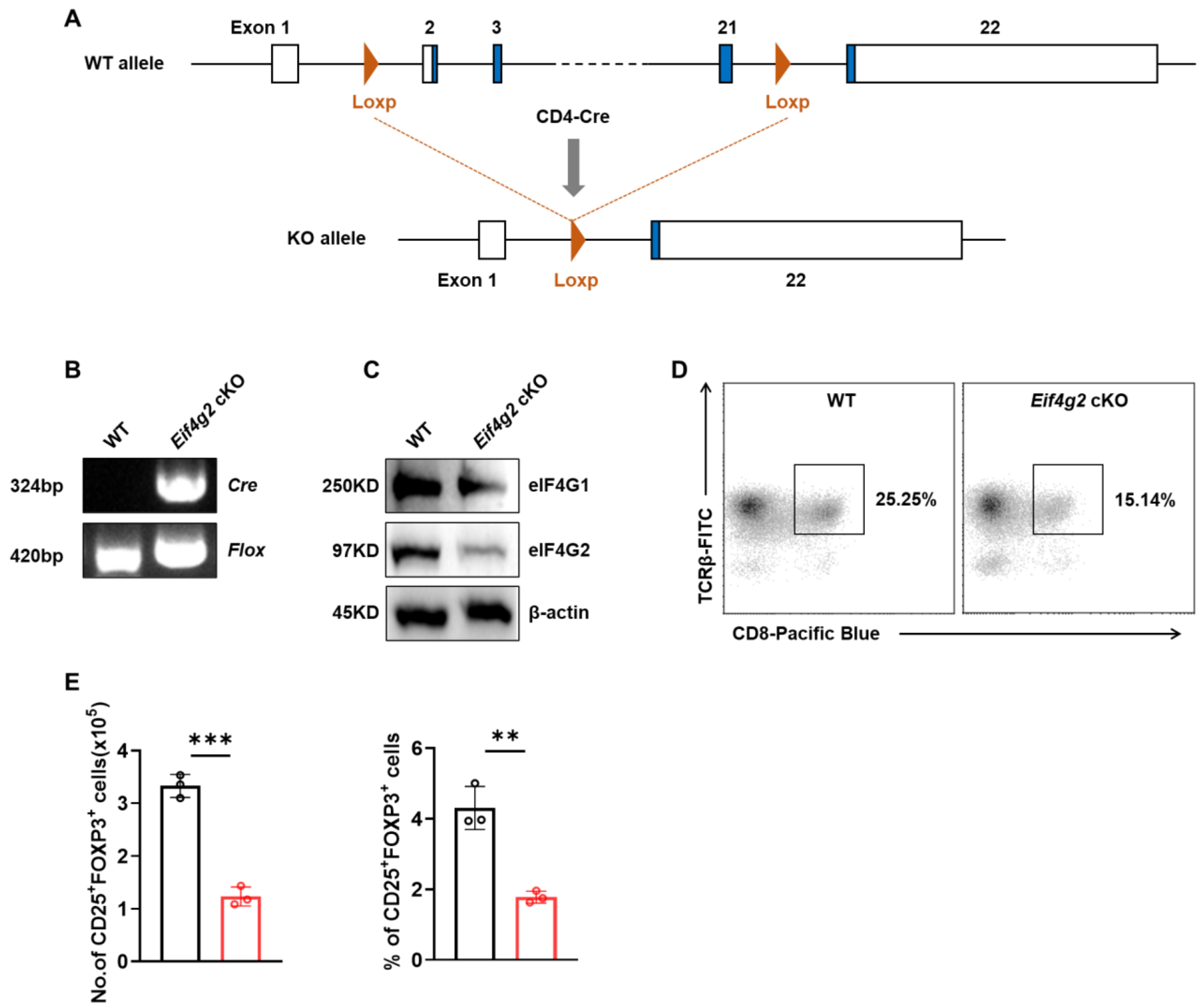

Figure S2. Characterization of *Eif4g2* cKO mice, related to Figure 2.

(A) Schematic of the generation strategy for *Eif4g2*$^{flox/flox}$ *Cd4-Cre* mice generation; (B) PCR genotyping of WT and *Eif4g2* cKO mice; (C) Western blot analysis for eIF4G1 and eIF4G2 protein level in thymocytes; (D) Gating strategy for identifying mature CD8 SP ($CD8^{+}TCR\beta^{+}$) cells; (E) Absolute number and frequency of mature Treg cells ($CD25^{+}Foxp3^{+}$) in $CD4^{+}$ T cells. n=3 mice per group, $^{**}p < 0.01$, $^{***}p < 0.001$. Data are representative of at least two independent experiments. Bar graphs show mean ± SEM and unpaired Students' t-test was used to perform the statistical analysis.

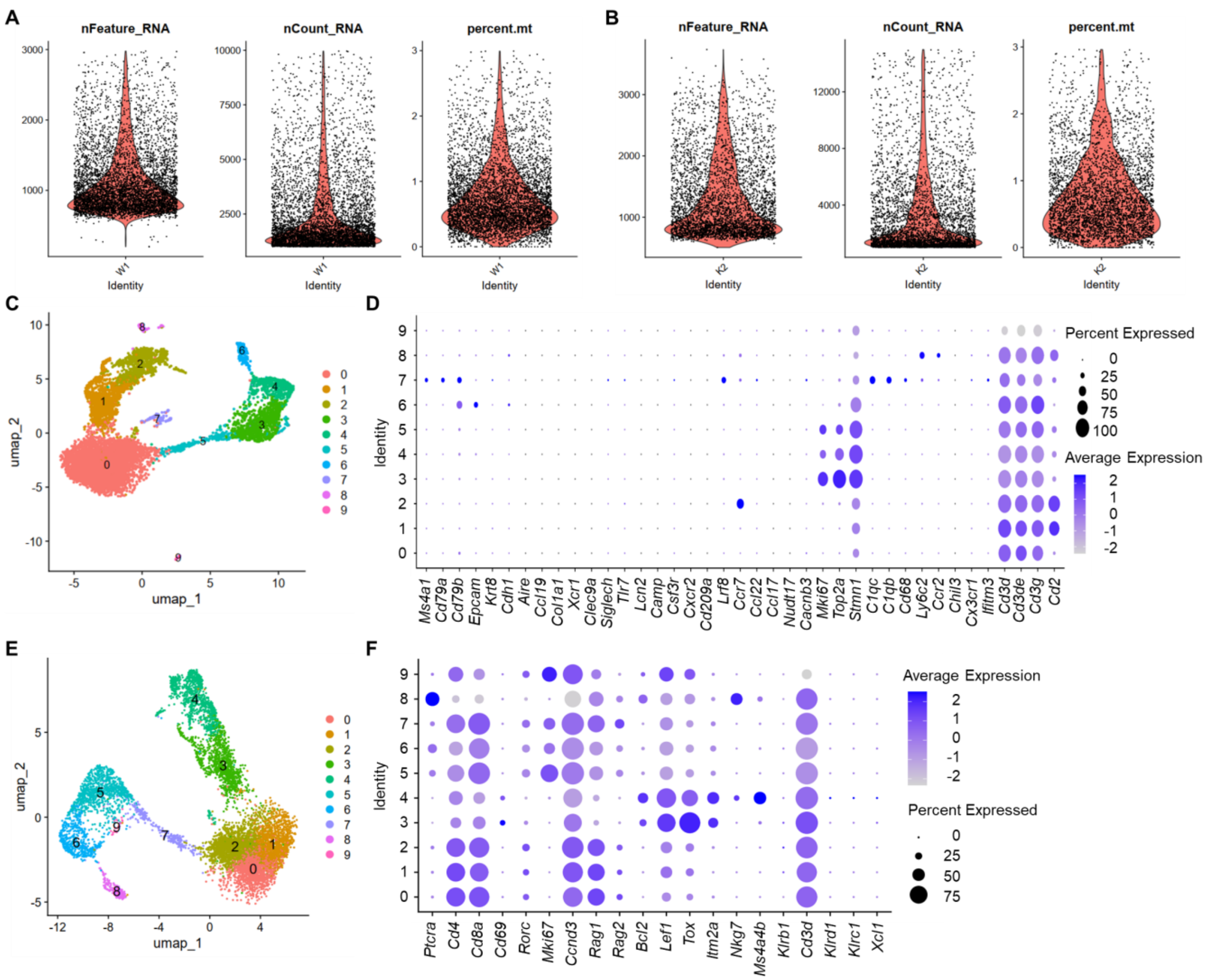

Figure S3. Quality control, unsupervised clustering, and annotation of thymic scRNA-seq data, related to Figure 4.

(A and B) Violin plots showing the distribution of quality control metrics (nFeature_RNA, nCount_RNA, and percent.mt) for cells from the W1 and K2 samples after applying filtering thresholds; (C) UMAP visualization of all thymic cells, colored by unsupervised clusters; (D) Dot plot showing the expression of canonical marker genes used to annotate the cell clusters in (C); (E) UMAP visualization of cells re-clustered from the T lineage subset, colored by sub-clusters. (F) Dot plot showing the expression of marker genes used to define the T-cell subpopulations in (E).

ID	Description	RichFactor	FoldEnrichment	zScore	pvalue	p.adjust	qvalue
mmu04660	T cell receptor signaling pathway	0.106557377	5.56292189	7.083445	5.6E-07	0.000144	0.00011
mmu04062	Chemokine signaling pathway	0.077319588	4.036537295	5.964735	4.58E-06	0.000589	0.000451
mmu05135	Yersinia infection	0.088235294	4.606401384	5.91499	1.12E-05	0.000958	0.000734
mmu04520	Adherens junction	0.097826087	5.107097187	5.528821	6.4E-05	0.003961	0.003034
mmu04070	Phosphatidylinositol signaling system	0.09375	4.894301471	5.35613	8.94E-05	0.003961	0.003034
mmu04670	Leukocyte transendothelial migration	0.084033613	4.387048937	5.192238	9.25E-05	0.003961	0.003034
mmu04510	Focal adhesion	0.064356436	3.359784508	4.731795	0.000135	0.00479	0.003669
mmu04611	Platelet activation	0.079365079	4.143323996	4.959964	0.000149	0.00479	0.003669
mmu04810	Regulation of actin cytoskeleton	0.059574468	3.110137672	4.570983	0.000169	0.004817	0.00369
mmu00310	Lysine degradation	0.109375	5.710018382	5.281304	0.00021	0.005405	0.00414
mmu04015	Rap1 signaling pathway	0.060465116	3.156634747	4.464205	0.00025	0.00583	0.004465
mmu04380	Osteoclast differentiation	0.073529412	3.83866782	4.655802	0.000279	0.005971	0.004573
mmu05132	Salmonella infection	0.055335968	2.888863055	4.249142	0.000361	0.006945	0.005319
mmu04928	Parathyroid hormone synthesis, secretion and action	0.077586207	4.050456389	4.616275	0.000378	0.006945	0.005319
mmu04666	Fc gamma R-mediated phagocytosis	0.085106383	4.443053817	4.685463	0.000429	0.007357	0.005635
mmu04917	Prolactin signaling pathway	0.094594595	4.938394277	4.750823	0.000519	0.008329	0.00638
mmu05205	Proteoglycans in cancer	0.058823529	3.070934256	4.173504	0.000552	0.00835	0.006395
mmu04517	IgSF CAM signaling	0.05	2.610294118	3.953576	0.000651	0.009302	0.007124
mmu04933	AGE-RAGE signaling pathway in diabetic complications	0.079207921	4.135119394	4.423883	0.000696	0.009409	0.007206
mmu04659	Th17 cell differentiation	0.076190476	3.977591036	4.284803	0.000899	0.011555	0.00885
mmu05161	Hepatitis B	0.06097561	3.183285509	3.937513	0.001211	0.014232	0.0109
mmu04931	Insulin resistance	0.072727273	3.796791444	4.120317	0.001218	0.014232	0.0109
mmu04658	Th1 and Th2 cell differentiation	0.079545455	4.152740642	4.150028	0.001461	0.015641	0.01198
mmu05235	PD-L1 expression and PD-1 checkpoint pathway in cancer	0.079545455	4.152740642	4.150028	0.001461	0.015641	0.01198
mmu04650	Natural killer cell mediated cytotoxicity	0.066666667	3.480392157	3.818487	0.002124	0.021474	0.016448
mmu05221	Acute myeloid leukemia	0.085714286	4.474789916	4.075954	0.002172	0.021474	0.016448
mmu05212	Pancreatic cancer	0.078947368	4.121517028	3.816342	0.003296	0.031369	0.024026
mmu05202	Transcriptional misregulation in cancer	0.048672566	2.540994274	3.272145	0.004157	0.038152	0.029221
mmu04010	MAPK signaling pathway	0.043478261	2.269820972	3.1123	0.004968	0.043665	0.033444
mmu03018	RNA degradation	0.072289157	3.773919206	3.545288	0.005097	0.043665	0.033444
mmu04662	B cell receptor signaling pathway	0.071428571	3.728991597	3.508981	0.005404	0.044798	0.034312
mmu04630	JAK-STAT signaling pathway	0.052631579	2.747678019	3.219542	0.005614	0.045085	0.034532
mmu04725	Cholinergic synapse	0.061403509	3.205624355	3.308572	0.006247	0.048654	0.037265
mmu05162	Measles	0.055172414	2.880324544	3.185762	0.006721	0.050806	0.038913
mmu04935	Growth hormone synthesis, secretion and action	0.05982906	3.123428859	3.227372	0.007179	0.052713	0.040374
mmu04072	Phospholipase D signaling pathway	0.053691275	2.803000395	3.097192	0.007877	0.056233	0.04307
mmu04664	Fc epsilon RI signaling pathway	0.075757576	3.954991087	3.365098	0.008525	0.058957	0.045156
mmu05032	Morphine addiction	0.064516129	3.368121442	3.205307	0.008801	0.058957	0.045156
mmu00514	Other types of O-glycan biosynthesis	0.093023256	4.856361149	3.540871	0.009	0.058957	0.045156
mmu04518	Integrin signaling	0.052287582	2.729719339	3.011503	0.009176	0.058957	0.045156
mmu05163	Human cytomegalovirus infection	0.04296875	2.243221507	2.81366	0.010288	0.064486	0.049392

Figure S4. Significantly altered pathways in *Eif4g2*-deficient thymocytes, related to Figure 4.

KEGG pathway enrichment analysis of genes dysregulated in *Eif4g2* cKO cells. Pathways are ranked by statistical significance.

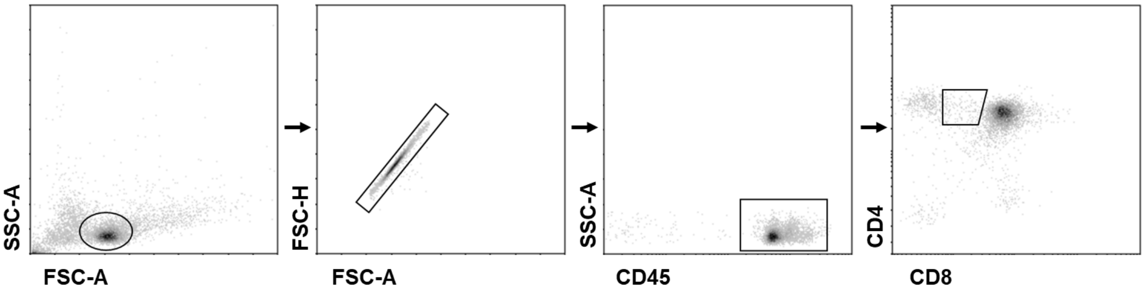

Figure S5. Isolation strategy for $CD4^{+}CD8^{lo}$ transitional cells, related to Figure 4.

Data S1. Unedited WB images and independent repeats

Related to Figure 2. A

Unedited images

Derived from the same gel

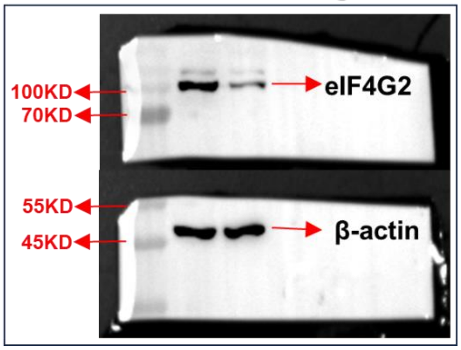

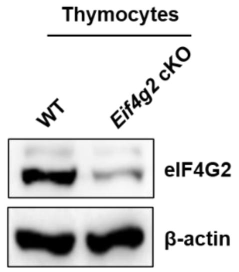

Independent repeats

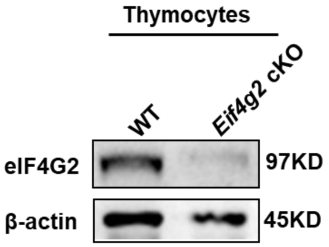

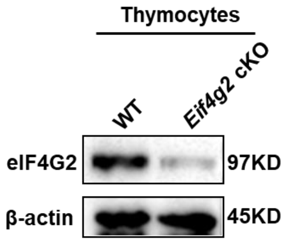

Related to Figure 2. B （left panel）

Unedited images

Derived from the same gel

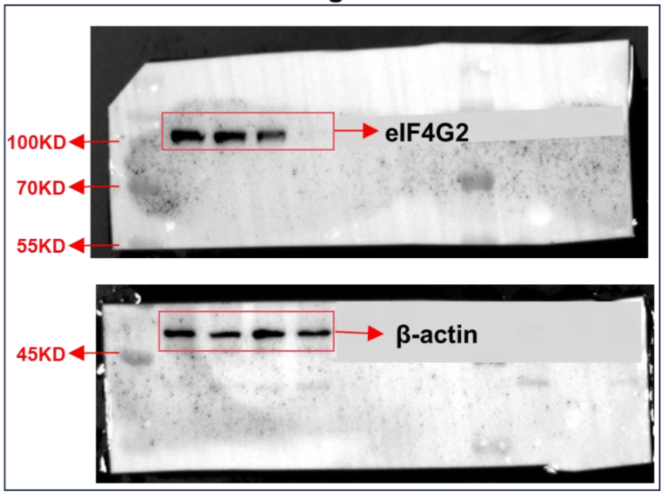

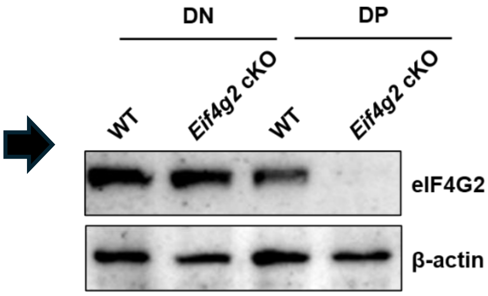

Independent repeats

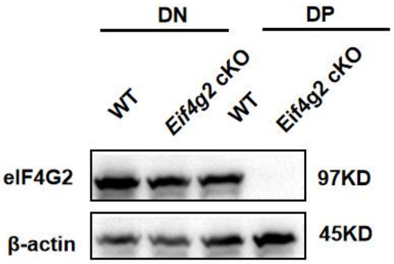

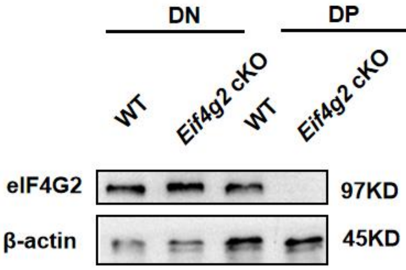

Related to Figure 2. B (right panel)

Unedited images

Derived from the same gel

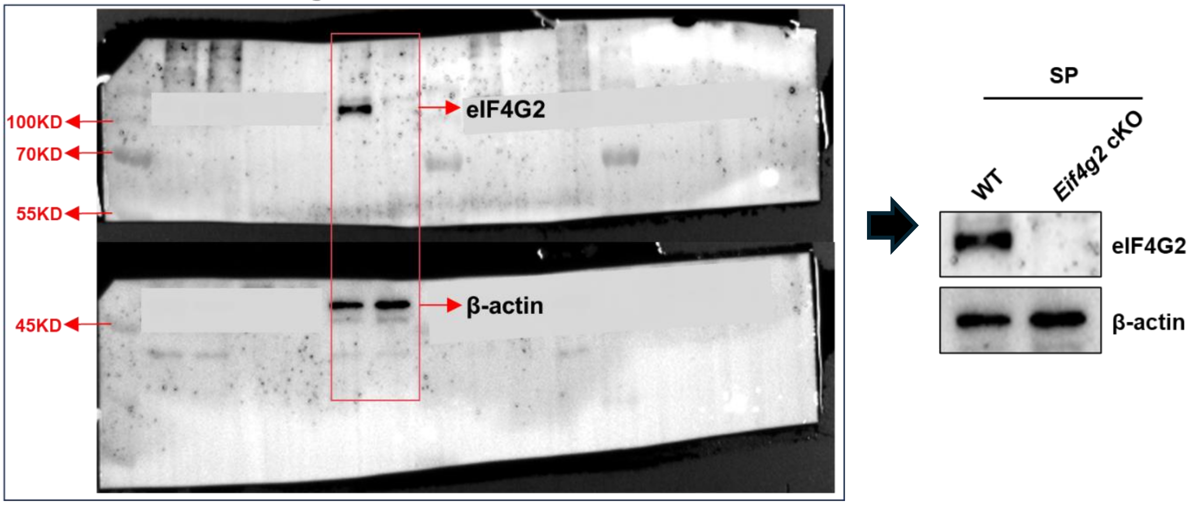

Independent repeats

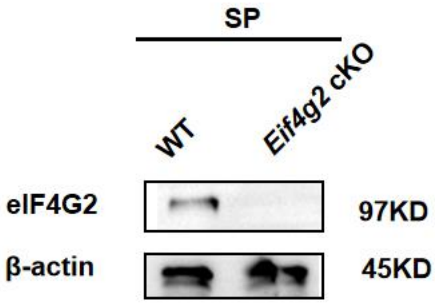

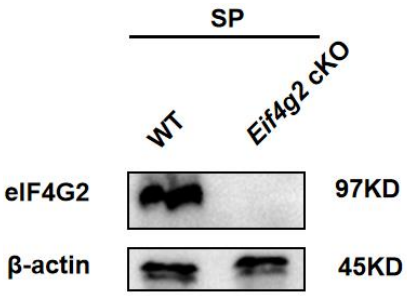

Related to Supplementary Figure 2. C

Unedited images

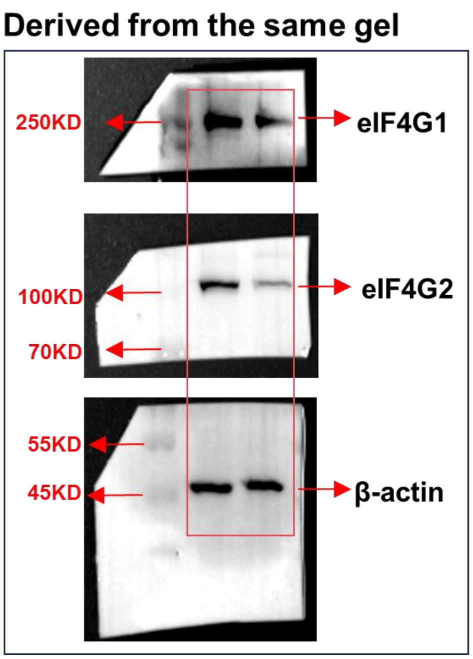

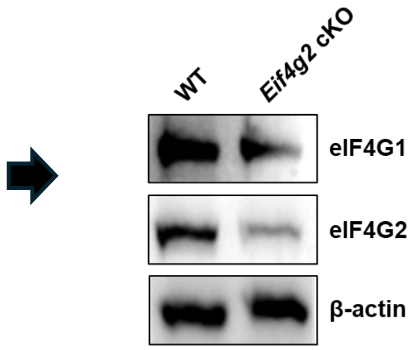

Independent repeats

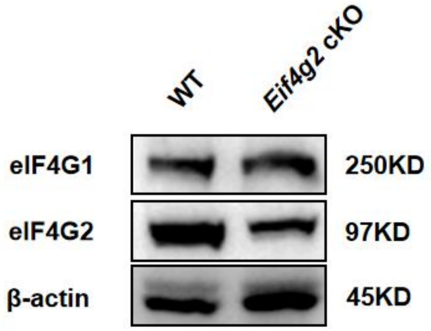

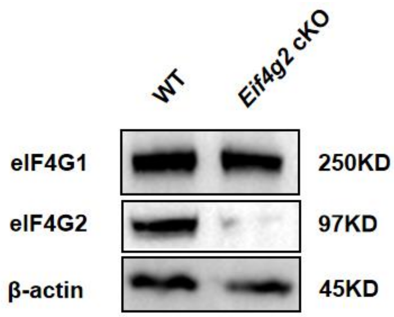

Related to Figure 4. F

Unedited images

Derived from the same gel

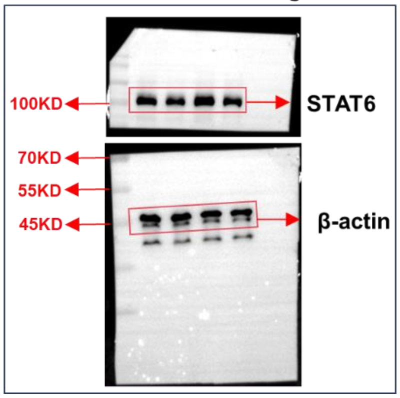

Separate but parallel gel

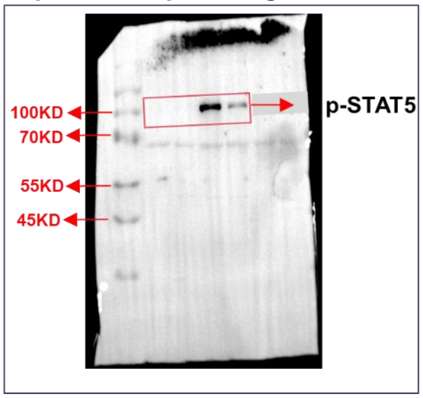

Separate but parallel gel

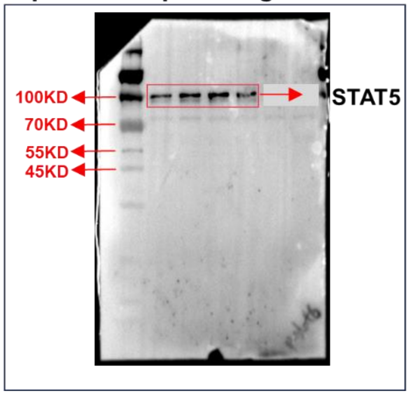

Separate but parallel gel

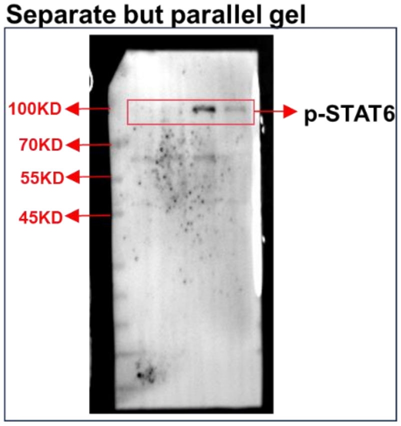

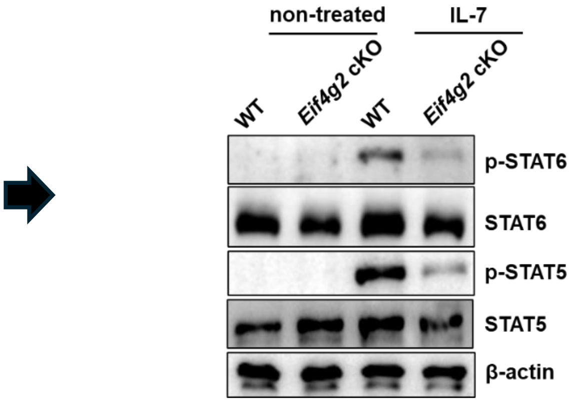

Independent repeats

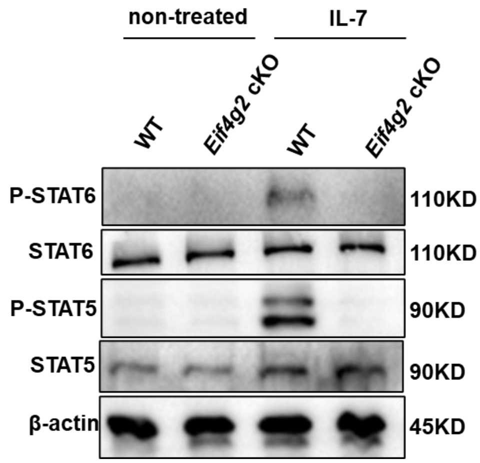

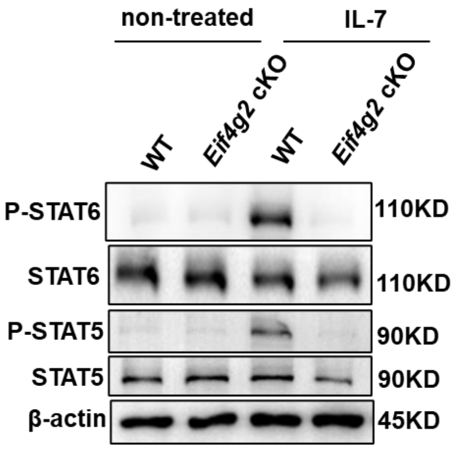

Related to Figure 6. A

Unedited images

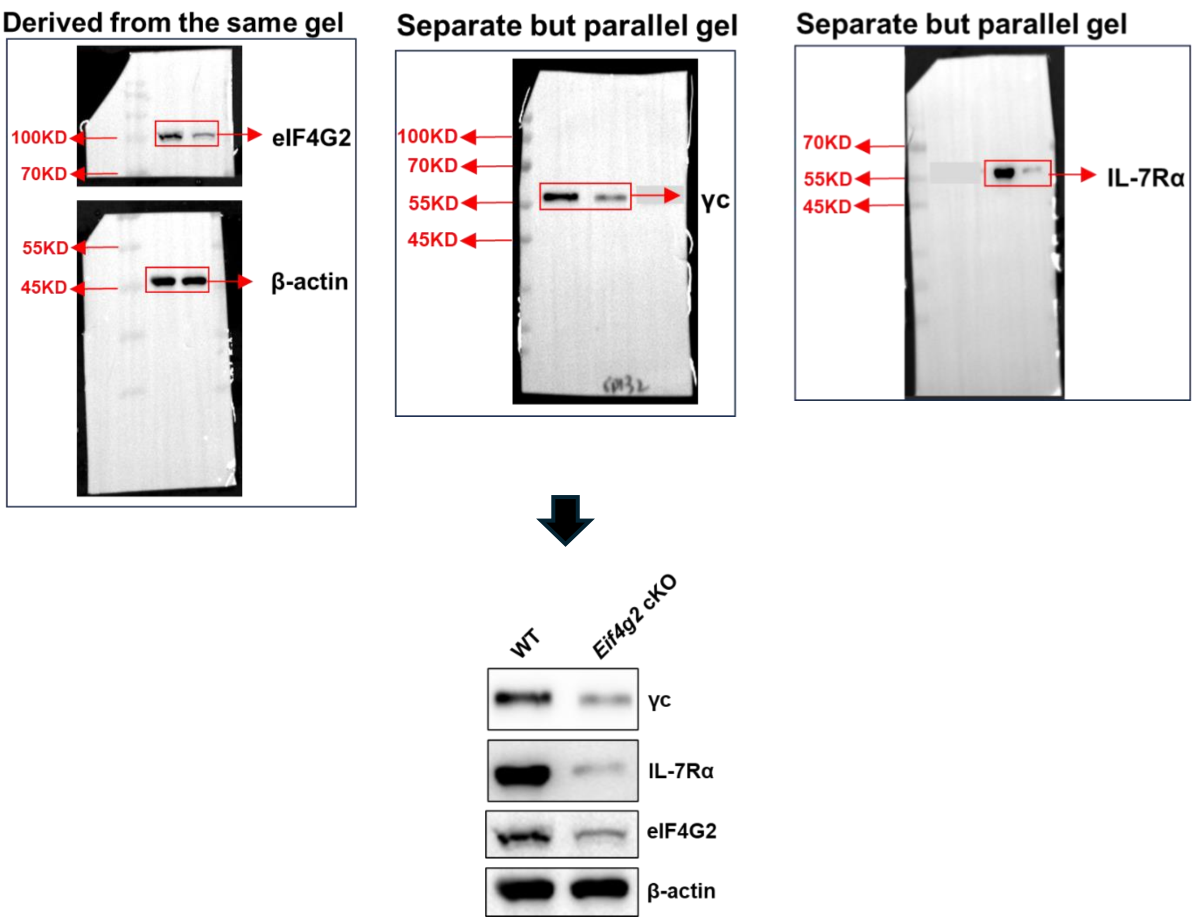

Independent repeats

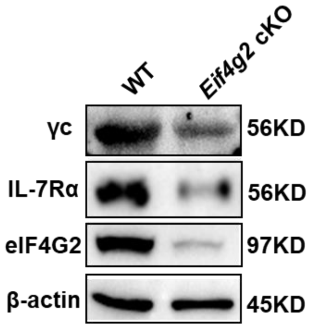

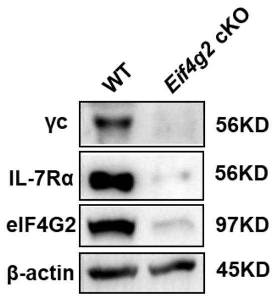

Related to Figure 6. D

Unedited images

Derived from the same gel   Separate but parallel gel

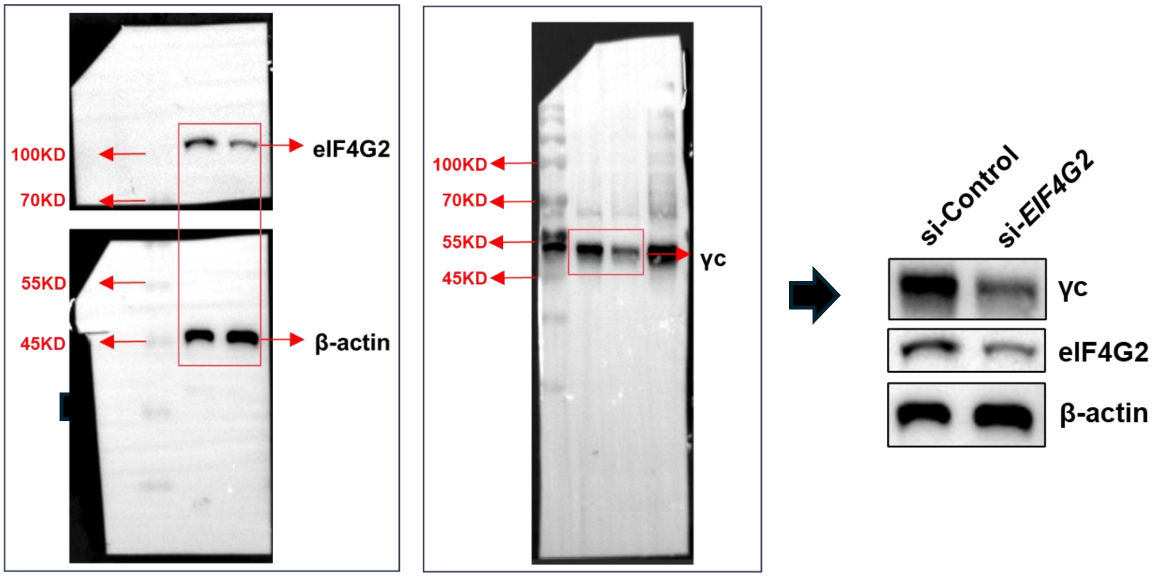

Independent repeats

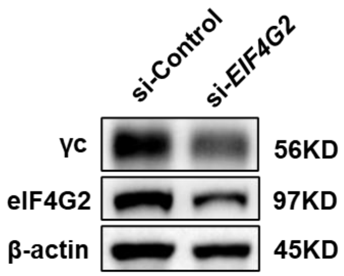

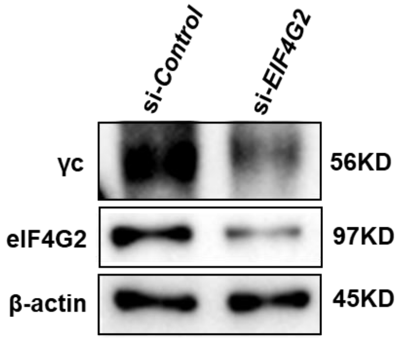

Related to Figure 6. F

Unedited images

Derived from the same gel

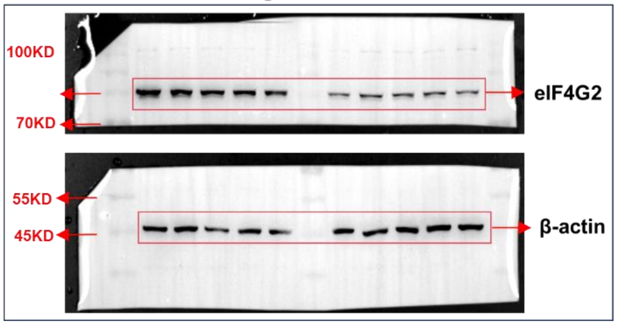

Separate but parallel gel

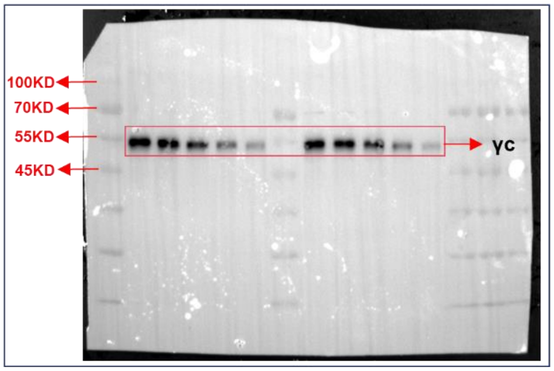

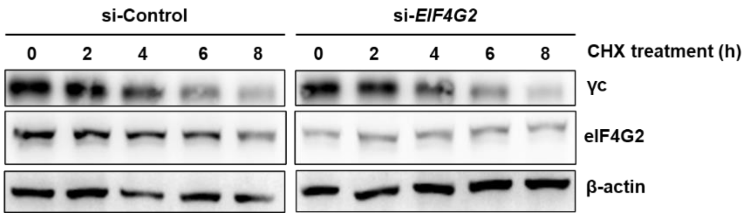

Independent repeats

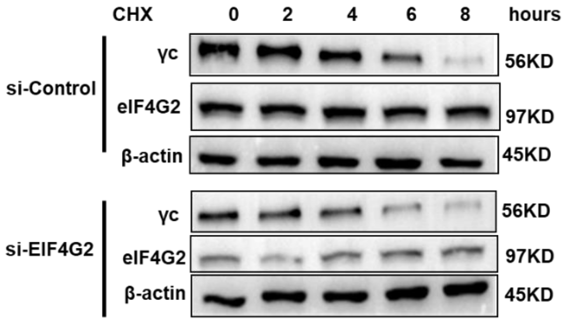

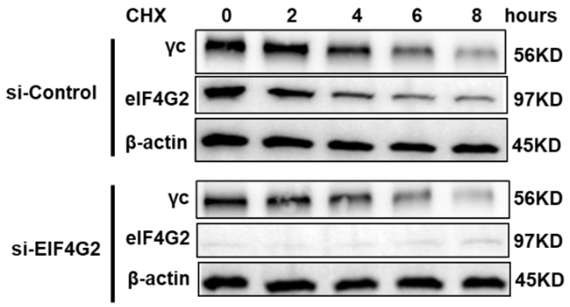

Related to Figure 6. H

Unedited images

Derived from the same gel

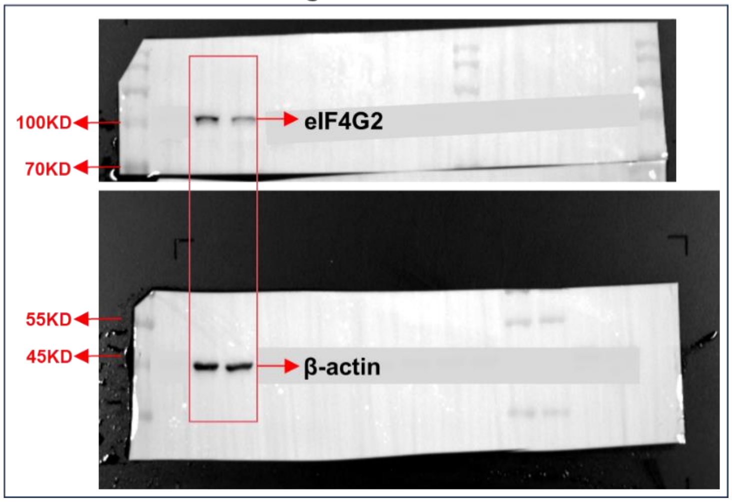

Separate but parallel gel

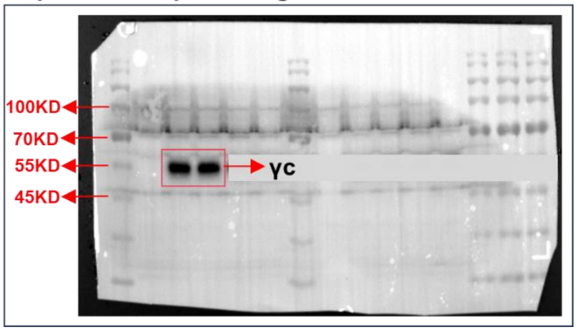

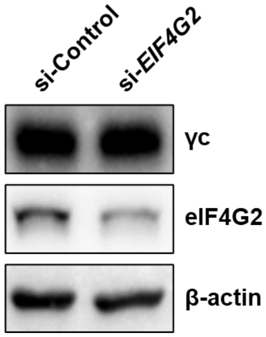

Independent repeats

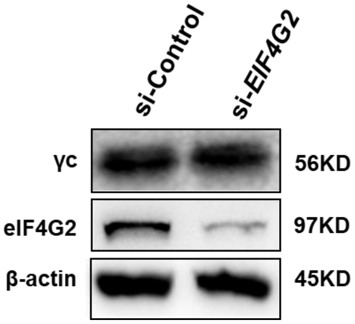

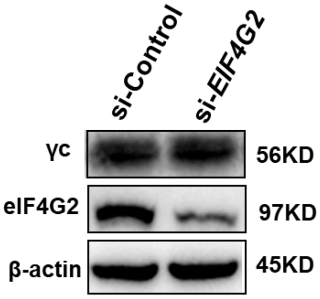

Supplement: Document S1. Figures S1-S5 and Unedited WB images and independent repeats [file mmc1.pdf]
